# Specific Features of Reactive Pulsed Laser Deposition of Solid Lubricating Nanocomposite Mo–S–C–H Thin-Film Coatings

**DOI:** 10.3390/nano10122456

**Published:** 2020-12-08

**Authors:** Vyacheslav Fominski, Dmitry Fominski, Roman Romanov, Mariya Gritskevich, Maxim Demin, Petr Shvets, Ksenia Maksimova, Alexander Goikhman

**Affiliations:** 1 National Research Nuclear University MEPhI (Moscow Engineering Physics Institute), Kashirskoe sh., 31, 115409 Moscow, Russia; dmitryfominski@gmail.com (D.F.); limpo2003@mail.ru (R.R.); mgritskevich@yandex.ru (M.G.); 2 Immanuel Kant Baltic Federal University, A. Nevskogo St 14, 236016 Kaliningrad, Russia; sterlad@mail.ru (M.D.); pshvets@kantiana.ru (P.S.); xmaksimova@gmail.com (K.M.); aygoikhman@gmail.com (A.G.)

**Keywords:** reactive pulsed laser deposition, solid lubricants, nanocomposite, molybdenum sulfides, coefficient of friction, wear, diamond-like carbon

## Abstract

This work investigates the structure and chemical states of thin-film coatings obtained by pulsed laser codeposition of Mo and C in a reactive gas (H_2_S). The coatings were analysed for their prospective use as solid lubricating coatings for friction units operating in extreme conditions. Pulsed laser ablation of molybdenum and graphite targets was accompanied by the effective interaction of the deposited Mo and C layers with the reactive gas and the chemical states of Mo- and C-containing nanophases were interdependent. This had a negative effect on the tribological properties of Mo–S–C–H nanocomposite coatings obtained at H_2_S pressures of 9 and 18 Pa, which were optimal for obtaining MoS_2_ and MoS_3_ coatings, respectively. The best tribological properties were found for the Mo–S–C–H_5.5 coating formed at an H_2_S pressure of 5.5 Pa. At this pressure, the *x* = S/Mo ratio in the MoS*_x_* nanophase was slightly less than 2, and the a-C(S,H) nanophase contained ~8 at.% S and ~16 at.% H. The a-C(S,H) nanophase with this composition provided a low coefficient of friction (~0.03) at low ambient humidity and 22 °C. The nanophase composition in Mo–S–C–H_5.5 coating demonstrated fairly good antifriction properties and increased wear resistance even at −100 °C. For wet friction conditions, Mo–S–C–H nanocomposite coatings did not have significant advantages in reducing friction compared to the MoS_2_ and MoS_3_ coatings formed by reactive pulsed laser deposition.

## 1. Introduction

Researchers and practitioners turned their attention to solid lubricating coatings based on transitional metal dichalcogenides (TMDs), such as MoS_2_, WS_2_, MoSe_2_, and WSe_2_, in the 1980s [1,2]. This was due to the need to improve qualitatively the tribological properties (low coefficient of friction, durability, wear resistance) of friction units operating in a vacuum or the inert environment of a spacecraft. By that time, sufficiently effective technologies for the deposition of such coatings (mainly ion sputtering) had already been developed. This made it possible to regulate flexibly the modes of deposition and the composition of the coatings [3,4]. At the initial stage of research into these coatings, the emphasis was on finding the optimal conditions for the deposition of monophase (pure) TMD coatings and for obtaining their required composition and structure. Soon the main problem of such coatings became apparent: it was low wear resistance, especially at high contact loads. Consequently, the focus of research shifted towards the search for nanocomposite and multilayer (nanolayer) coatings. The wear resistance of nanocomposite coatings can be significantly increased by combining the plastic TMD phase with a harder/stronger or corrosion-resistant component (metals, hard carbon, metal carbides or nitrides) [5,6,7,8].

Despite the breakthroughs in the formation of nanocomposite TMD-based coatings with improved tribological characteristics, the problem of obtaining solid lubricating coatings to reduce friction under various operating conditions remains topical. This is due to both the growing demand for such coatings in traditional and new hi-tech industries (space technology, vacuum/cryogenic technology, micromechanics, etc.), and the interest in new processes that can bring about a radical change in the patterns of friction and wear. Modern studies show that it is necessary to regulate the architecture of the coating, i.e., morphology and phase composition, at the nanoscale to improve the tribological properties of nanocomposite TMD-based coatings [9,10,11,12,13,14]. In this case, triboinduced processes in the contact layer can change significantly and new nanophases can form in the tribofilm.

Nanocomposite coatings containing the TMD nanophase and hard carbon/diamond-like carbon/graphene have always stirred the intense interest of researchers since the C-based nanophase causes both the strengthening of the coating and contributes to the manifestation of the “chameleon” effect upon changing friction conditions [5,9,15]. In high air humidity, nanocomposite coatings can be used for a wider array of applications. During triboactivated interaction of nanodiamonds with the ultrafine (2D) MoS_2_ phase, onion-like inclusions can form in the tribolayer. This leads to a decrease in the friction coefficient in vacuum to very low values (~0.005) [16]. Certain composition of coatings containing nanosized MoS_2_ phases and diamond-like carbon demonstrate superlubricity: reduced wear can be achieved in air due to the formation of a tribofilm containing nanoscrolls of graphene-like material [17]. Many researchers note a significant effect of such graphene-like carbon nanoscrolls on friction and wear [18,19].

The majority of works on the production and study of nanocomposite TMD-based films analyse the method of deposition by ion (magnetron) sputtering of multisector targets. The method of ion sputtering has been used in many experiments and is still being perfected [20,21]. Pulsed laser deposition (PLD) is also used to form sufficiently high-quality solid lubricating TMD-based coatings [22,23,24,25]. This method differs from the more traditional magnetron sputtering since PLD gives precise control of the growth rate of the coating (up to one monolayer of the material) [26]. It allows researchers to obtain numerous combinations of different materials under controlled vacuum conditions; it is also possible to supplement deposition by the implantation of high-energy ions for ion mixing [27,28]. Yet, the PLD method has a deficiency due to the specificity of pulsed laser ablation of TMD targets. It is difficult to achieve the deposition of pure vapour (plasma) during the ablation of MoS_2_ targets prepared by pressing MoS_2_ powder. The ablation of the MoS_2_ target results as a rule in the formation of microparticles/microdroplets, the deposition of which contributes to the formation of the porous structure of the coating [29,30]. During pulsed laser ablation of MoSe_2_ and WSe_2_ targets, explosive boiling of the material and the formation of liquid metal droplets can occur. However, for these materials, most of the particles on the surface of the coating are round in shape and ~10–100 nm in size [31,32,33]. Such nanoparticles do not have any noticeable negative effect on the formation of high-quality monophase and nanocomposite coatings with solid lubricating components MoSe*_x_* and WSe*_x_* [24,27,33]. Still, these nanoparticles may complicate the formation of multilayer coatings with layer thickness controlled at the nanoscale [34].

The aim of this work is to study the composition, structure, and tribological properties of Mo–S–C–H thin-film coatings formed by reactive PLD (RPLD) on steel substrates at room temperature. Reactive PLD makes it possible to form smoother and more uniform layers of a molybdenum sulphide MoS*_x_* and alter the ratio of elements *x* = Mo/S over a wide range (1 ≤ *х* ≤ 4) [35]. Varying the composition of the coatings has proven to be an important factor for the development of their specific applications. For instance, the MoS_3_ coatings of clustered type demonstrated improved antifriction properties when tested at low temperatures (−100 °C) and low humidity. The deficiency of these coatings is their reduced wear resistance compared to MoS_2_ coatings. As shown above, the wear resistance of such coatings can be improved through the formation of a nanocomposite material containing both the MoS*_x_* nanophase and the nanophase of hard (diamond-like) carbon. During the RPLD of a Mo–S–C–H nanocomposite coating from a target containing sectors of pure molybdenum and graphite, the deposition of a pulsed laser plume occurs in a reactive medium—hydrogen sulphide (H_2_S). Fominski et al. [35] revealed the dependence of the composition of solid lubricate MoS*_x_* thin-film coatings on the pressure of H_2_S. The influence of H_2_S on the composition and properties of carbon thin films obtained by RPLD remains unexplored, which creates difficulties in choosing optimal conditions for obtaining high-quality nanocomposite coatings Mo–S–C–H.

It was found in the work that during the ablation of graphite target in H_2_S, the deposited a-C(S,H) films effectively captured S and H atoms. The tribological properties of nanocomposite Mo–S–C–H coatings under various sliding conditions were largely determined by the properties of amorphous a-C(S,H) nanophases, which strongly degraded with an increase in S content. The Mo–S–C–H coating formed at a relatively low H_2_S pressure (~5.5 Pa) turned out to be the most promising. Overall, these coatings outperformed MoS_2_ and MoS_3_ monophase coatings in their tribological characteristics when tested in dry friction conditions at room and low (−100 °C) temperatures. In humid air at room temperature, the Mo–S–C–H coatings did not show a noticeable improvement of low friction properties in comparison with the MoS_2_ and MoS_3_ monophase coatings previously obtained by RPLD and studied in [35].

At first glance, the RPLD technique is not quite suitable for large area deposition, especially onto shaped work pieces used in practice. Moreover, the use of H_2_S gas requires special safety measures since it is explosive and toxic. However, several important issues should be highlighted in the research of this technique. Within the framework of a fundamental problem, if new nanomaterials with unique/interesting properties would be formed by RPLD, these results could initiate improving more conventional deposition techniques (e.g., ion-sputter deposition). From a practical point of view, the solvent of environmental safety problem arising due to H_2_S is not very complicated. In the case of the unique results of the RPLD application, the area of the covered surface can be increased by moving/rotating the processed parts under a laser-induced plume. Also, the use of pulsed electric fields applied to a shaped work piece would improve the uniformity of RPLD treatment. Obviously, the RPLD technique can be applied (and even difficult to replace) when processing small-sized parts or the inner surface of pipes/rings.

## 2. Materials and Methods

Reactive pulsed laser deposition technique for MoS*_x_* thin-film coating formation was analysed in detail in [35]. The Mo target was ablated by nanosecond laser pulses with a radiation wavelength of 1064 nm. The pulse energy did not exceed 85 mJ at a pulse repetition rate of 25 Hz. A laser fluence of ~20 J/cm^2^ ensured efficient evaporation of the Mo target without any noticeable formation of a droplet fraction. When obtaining Mo–S–C–H films, a graphite target was placed next to the Mo target. The ablation time of the Mo target was 8 s, and the C target—4 s. Before the ablation, the film deposition chamber was evacuated with a vacuum pump to a pressure no higher than 10^−3^ Pa. Then, hydrogen sulphide was introduced into the chamber to a predetermined pressure. The pressures were chosen taking into account the results of [35]. Fominski et al. [35] established that for obtaining films MoS_1.5_, MoS_2_ and MoS_3_, the pressure of hydrogen sulphide had to be maintained at 5.5, 9, and 18 Pa, respectively. The prepared nanocomposite coatings were designated taking into account the H_2_S pressure used: Mo–S–C–H_5.5, Mo–S–C–H_9 and Mo–S–C–H_18.

To reveal the effect of H_2_S on the carbon nanophase, additional experiments on the deposition of carbon films from a graphite target at the same H_2_S pressures were carried out. To better understand the effect of hydrogen sulphide on the deposition rate of Mo and C, Mo‒C films were obtained under vacuum conditions (at a residual gas pressure of 10^−3^ Pa).

We used polished discs made of 95Cr18 stainless steel (C content of 0.95% and Cr content of 18%) and polished silicon wafers as substrates for the deposition of thin-film coatings. The total deposition time of the Mo–S–C–H coatings on the steel substrates was 40 min and on silicon substrates, 20 min. The total thickness of Mo–S–C–H thin-film coatings on steel substrates was ~300‒400 nm.

The substrates were kept at room temperature during the formation of the coatings. Before the deposition of the Mo–C–S–H coatings on the steel substrates, a SiC sublayer was deposited on the surface of the substrates. The sublayer was obtained by PLD from the SiC target under vacuum conditions. It was considered that the silicon carbide sublayer can increase the adhesion of C-based coatings to the steel substrate [36,37].

To determine the atomic composition of the coatings, Rutherford back-scattering spectroscopy (RBS) and elastic recoil detection analysis (ERDA) techniques were used. The energy of helium ions in the analysing beam was 1.5 MeV, and the detector resolution was 20 keV. The RBS spectra were recorded in the configuration α = 0°, β = 20°, θ = 160°. The ERDA spectra were recorded in the configuration α = 80°, β = 80°, θ = 20°. The measured spectra data were processed using the Simnra software (Max-Planck-Institut für Plasmaphysok, Garching bei München, Germany). The surface morphologies of the Mo–S–C–H coatings were studied using scanning electron microscopy (SEM, Tescan LYRA 3, Brno, Czech Republic) before and after friction testing. The crystal structure of the coatings was examined by grazing incidence X-ray diffraction (XRD) using an angle of 5° and Cu Kα radiation in an Ultima IV (Rigaku, Tokyo, Japan) diffractometer. The chemical states of the coatings were studied by X-ray photoelectron spectroscopy (XPS). The XPS spectra were obtained by a Theta Probe Thermo Fisher Scientific spectrometer (Madison, WI 53711, USA) with a monochromatic Al Kα X-ray source (1486.7 eV) and an X-ray spot size of 400 μm. The photoelectron take-off angle was 50° with respect to the surface plane. The spectrometer energy scale was calibrated using Au4f7/2 core level lines located at E = 84.0 eV.

The structure of the coatings before and after the friction tests was studied by micro-Raman spectroscopy (MRS). Raman spectra of the samples were collected using a Horiba Jobin Yvon micro-Raman spectrometer LabRam HR800 (Horiba, Kyoto, Japan) with a 100× magnification lens. Measurements were conducted at room temperature in air. A He-Ne laser with a 632.8 nm wavelength was used to excite Raman scattering. The irradiation power density on the sample was chosen to avoid any structural changes or phase degradation in the films. The typical measurement conditions involved a laser power of ~1 mW and a laser spot with a diameter of ~30 μm.

To study the structure of the nanocomposite coatings at the nanoscale level, thin Mo–S–C–H films were deposited on NaCl substrates. The conditions for obtaining thin Mo–S–C–H films reproduced the conditions for obtaining coatings on steel discs. Thin films were studied using transmission electron microscopy (TEM, including high-resolution HRTEM) and selected area diffraction (SAED) in a JEM-2100 microscope (JEOL Ltd., Tokyo, Japan). The films deposited on NaCl crystals were first planted in water using a metal mesh and then transferred to the microscope to obtain a planar image.

The friction testing of thin-film coatings was carried out with the help of an Anton Paar TRB3 tribometer (Anton Paar GmbH, Graz, Austria) in the reciprocating motion mode, using a steel ball (100Cr6) with a diameter of 6 mm as a counterbody. The load on the ball was 1 N, and the Hertzian contact stress was ~660 MPa. The average speed of the ball over a substrate with a Mo–S–С–H coating was 1cm/s. The length of the wear track was 5 mm. A detailed description of the technique and setup for friction testing can be found in [35]. Three conditions were selected for testing, differing in ambient humidity and substrate temperature. The first tests were carried out at 22 °C in air at a related humidity (RH) ~58% (wet friction conditions). The second tests were carried out at a reduced atmospheric humidity (RH ~8%, dry friction condition), which was achieved by pumping argon through the testing chamber. The sample temperature was 22 °C. The third series of tests was carried out at low humidity (RH ~8%) and the sample was cooled to −100 °C (low temperature/dry friction conditions). The wear tracks were studied by MRS, SEM, optical microscopy, and optical profilometry.

## 3. Results

### 3.1. Composition of Mo–S–C–H Films Obtained by RPLD

**Figure 1:**
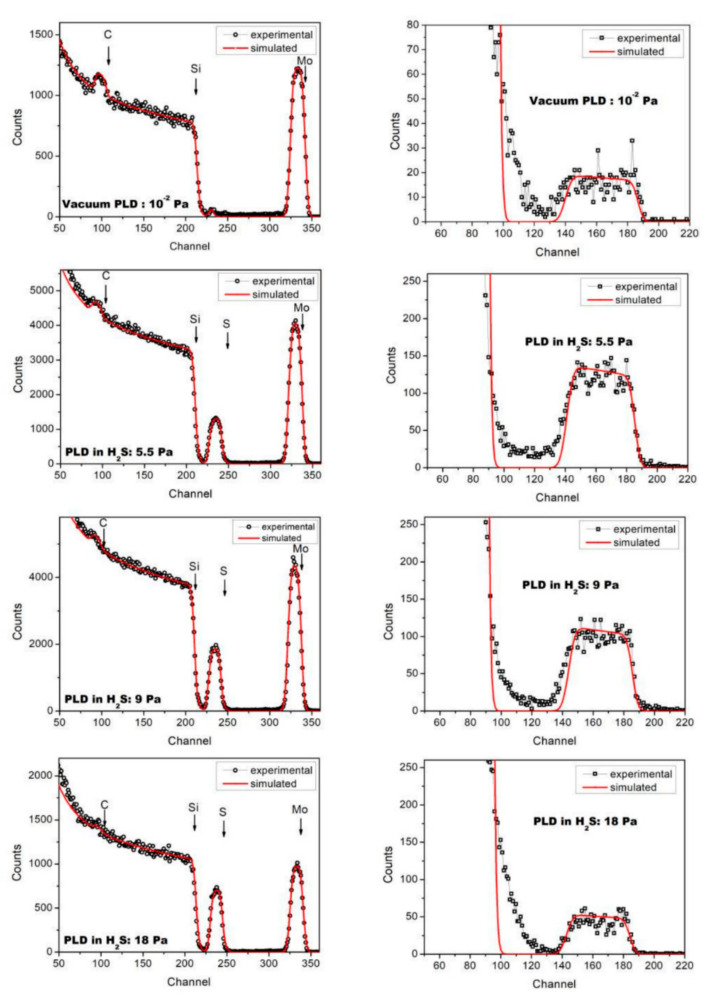
Experimental and simulated RBS (**left**) and ESDA (**right**) spectra of the films prepared on Si substrates by pulsed laser co-deposition of carbon and molybdenum under vacuum conditions (residual gas pressure was ~10^−3^ Pa) and in H_2_S gas with different pressures. The RBS spectrum of the film deposited in vacuum contains a peak at the channel number 230. This peak is due to scattering of ions by sulfur atoms that have been adsorbed on the surface of the Si substrate before the film deposition.

Figure 1 shows the experimental and simulated RBS and ERDA spectra for Mo–C and Mo–S–C–H films obtained by pulsed laser codeposition of molybdenum and carbon under vacuum conditions and in H_2_S gas with different pressures. For the film obtained under vacuum, mathematical processing of the spectra showed that the composition of this film was described by the formula C_0.81_Mo_0.16_H_0.03_. There was almost no sulphur in the bulk of Мo–С–H film. A small amount of sulphur was found at the boundary of the Mo–C film with the Si substrate and on the surface of the Mo–C–H film. This was possibly due to the fact that the walls of the deposition chamber were not subjected to any special treatment prior to the formation of the Mo–C–H films. The walls of the chamber were covered with a thin S-containing film, formed during previous experiments on RPLD of MoS*_x_* films. Sulphur atoms desorbing from the walls of the chamber could have deposited on the surface of the Si plate during vacuum pumping of the chamber and on the surface of the Mo–C–H film—after its deposition and storage (for some time) in the chamber. The presence of a small amount of hydrogen in the Mo–C–H films was possibly due to the interaction of the growing film with residual water vapour in the deposition chamber.

According to the RBS data, the thickness of the Mo–C–H film obtained during 20 min of deposition was 8.2 × 10^17^ atom/cm^2^. In reactive H_2_S gas, S atoms penetrated the deposited Mo–C–S–H film. Despite this fact, the overall deposition rate of atoms decreased. For hydrogen sulphide pressures of 5.5, 9, and 18 Pa, the composition of the films was described by the formulas C_0.49_Mo_0.13_S_0.28_H_0.1_, C_0.405_Mo_0.13_S_0.37_H_0.095_, and C_0.24_Mo_0.115_S_0.55_H_0.095_ respectively. The thickness of these films was 6.2 × 10^17^, 5.8 × 10^17^, and 5.2 × 10^17^ atom/cm^2^. For ablation of graphite and molybdenum targets, 1.5 × 10^4^ laser pulses were used, which were divided into series of 100 pulses for the C target and 200 pulses for the Mo target. The ablation of the C target resulted in the deposition of ~2 × 10^15^ atom/cm^2^ in hydrogen sulphide. This was sufficient for the formation of approximately one monolayer of amorphous carbon, given the atomic density in amorphous carbon of ~(1 ÷ 1.7) × 10^23^ atom/cm^3^. The same estimates for Mo showed that a 0.6 ÷ 1 MoS*_x_* monolayer could be formed after 200-pulse ablation of the Mo target in hydrogen sulphide.

**Figure 2:**
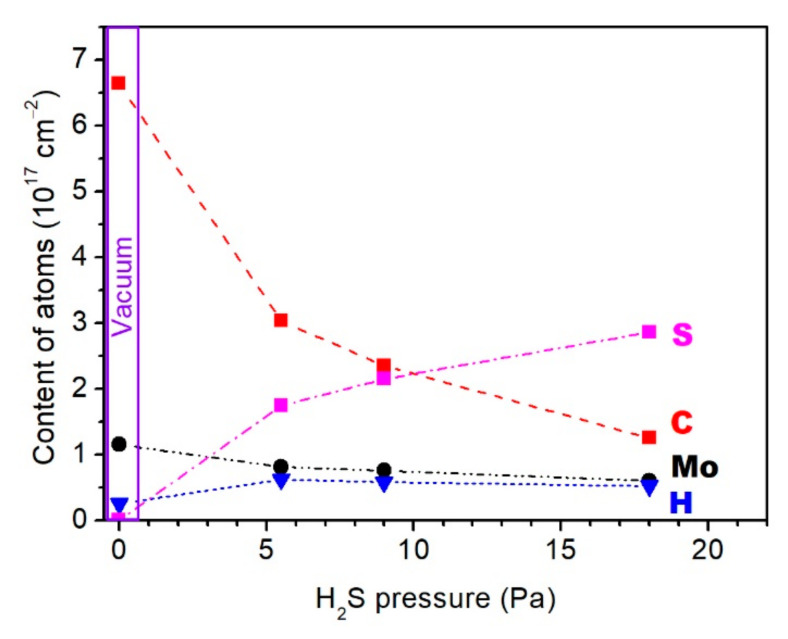
Influence of hydrogen sulfide pressure on the composition of Mo-S-C-H films which were obtained by pulsed laser codeposition of carbon and molybdenum in 20 min.

Figure 2 shows a change in the rate of deposition of various elements in the Mo–C–H and Mo–S–C–H films following an increase in the hydrogen sulphide pressure. As it is shown, H_2_S gas did not only ensure the saturation of the films with S atoms, but also had a significant effect on the rate of carbon deposition. At a pressure of 18 Pa, the rate of carbon deposition dropped almost fivefold compared to the rate of deposition in a vacuum. Under the same conditions, the rate of deposition of Mo atoms decreased only twofold. This could be explained by the difference of collisions when light (C) and heavy (Mo) atoms moved through H_2_S gas. The relatively heavy Mo atoms changed their trajectory only slightly and slowly lost their kinetic energy in collisions with the relatively light H_2_S molecules. When colliding with the H_2_S molecules, the lighter C atoms are scattered at large angles and leave the area where the deposition on the substrate took place. The possibility of reactive collisions of carbon ions with H_2_S molecules cannot be ruled out. These collisions may have resulted in the formation of volatile hydrocarbon molecules. This is a possible explanation as to why an increase in the H_2_S pressure did not result in a discernible change in the rate of the saturation of the films with hydrogen.

The analysis of the RBS and ERDA data for a-C(S,H) films formed during pulsed laser ablation of graphite in H_2_S confirmed the assumption about the effective interaction of the ablated flux of carbon atoms and H_2_S molecules (see Figure S1, Supplementary Materials). An increase in the pressure of hydrogen sulphide resulted in a noticeable increase in the concentration of sulphur in the a-C (S, H) films. The compositions of the films at hydrogen sulphide pressures of 5.5, 9, and 18 Pa were described by the formulas C_0.71_S_0.15_H_0.14_, C_0.61_S_0.26_H_0.13_, and C_0.42_S_0.4_H_0.18_. The thicknesses of the films were 2.2 × 10^18^, 1.7 × 10^18^ and 1.0 × 10^18^ atom/cm^2^ respectively. During the PLD of carbon films in vacuum (residual gas pressure ~10^−3^ Pa), their thickness was 2.88 × 10^18^ atom/cm^2^, and the H atoms concentration did not exceed 1 at.%.

### 3.2. Morphology, Structure, and Chemical State of Mo–S–C–H Films Obtained by RPLD

**Figure 3:**
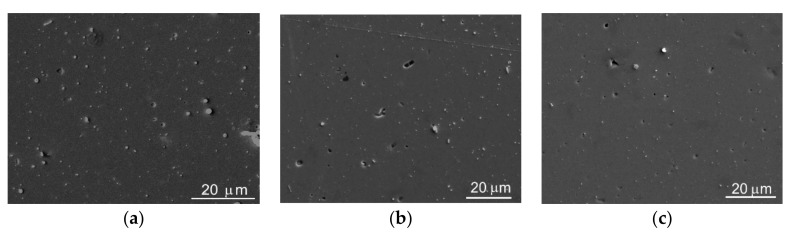
SEM images of the surface of Mo–S–C–H thin-film coatings obtained by reactive pulsed laser deposition (PLD) on steel substrates at the following H_2_S pressures: (**a**) 5.5; (**b**) 9; (**c**) 18 Pa.

The use of RPLD for obtaining Mo–S–C–H thin films made it possible to produce sufficiently smooth and dense coatings, the morphology of which, according to the results of SEM studies (Figure 3), did not depend on the H_2_S pressure. There were individual round-shaped particles of a submicron size on the surface of the Mo–S–C–H coatings. These particles could have formed because of the deposition of droplets formed during the ablation of the Mo and graphite targets. Such particles were found in a-C(S,H) films produced by RPLD from a graphite target in H_2_S gas (Figure S2, Supplementary Materials).

**Figure 4:**
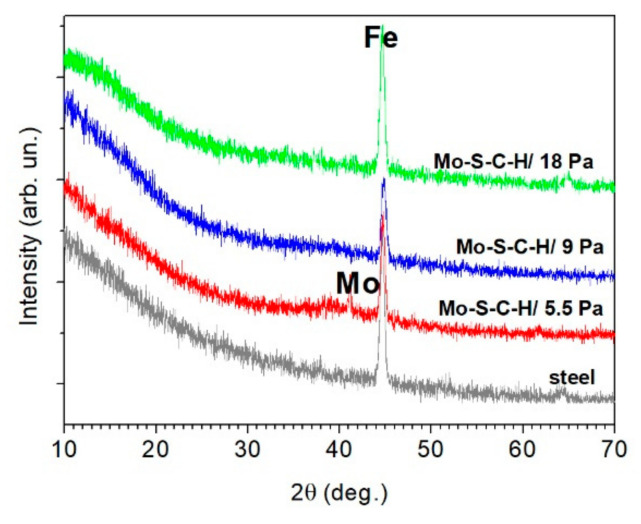
In plane grazing incidence X-ray diffraction patterns of Mo-S-C-H thin-film coatings obtained on steel substrates by the reactive PLD at various pressures of H_2_S gas. For comparison, X-ray diffraction pattern for the bare steel substrate is shown.

The results of the XRD of Mo–S–C–H thin-film coatings (Figure 4) show the deposition of Mo particles upon ablation of the Mo target. The X-ray diffraction pattern of the Mo–S–C–H_5.5 coating had a weak intensity peak, which corresponded to the (110) reflection for a body-centred cubic Mo lattice. The peak was practically invisible in the X-ray diffraction patterns of the Mo–S–C–H_9 and Mo–S–C–H_18 coatings. This could be attributed to the fact that with an increase in the pressure of H_2_S gas, the surface of the Mo target interacted with the reactive gas. Following this interaction, molybdenum sulphides could form on the target surface; this changed to a certain extent the mechanism of pulsed laser ablation of the Mo target.

XRD studies showed that at all selected pressures of H_2_S, the Mo–S–C–H thin-film coatings had an amorphous structure with a broadened diffraction peak in the angle range from 35° to 50°. With greater hydrogen sulphide pressure, the intensity of this peak noticeably weakened. This indicated an increased disordering of the structure following a rise in the sulphur concentration. This type of XRD pattern has been extensively described in the literature; in most experiments, the Mo–S–C coatings have been obtained by ion sputtering/codeposition from MoS_2_ and graphite targets (for example, [15,38,39,40]). For TMD coatings having an amorphous structure, this broad peak is usually explained by the formation of nanosize inclusions with a hexagonal lattice of the 2H-MoS_2_ type [41]. In the cases when there was no peak at angles 2θ~13°, but there was a peak in the 2θ range from 35° to 50°, the turbostratic stacking of (10*L*) planes into Type I texture was supposed. With this texture, the basal planes (002) are oriented perpendicular to the surface of the substrate [42]. The absence of a peak at 2θ~13° shows that the reactive PLD of Mo–S–C–H_5.5 films in H_2_S may not have caused the formation of a self-assembled multilayer structure MoS*_x_*/a-C (doped with Mo/S/H) with a periodicity in the nanometer scale, as it was the case during the magnetron sputtering of graphite and MoS_2_ targets in Ar/N_2_ gases [14,39]. In XRD patterns for the Mo–S–C–H_18 coatings, a weak-intensity and a very broad band appears at 2θ~15°. This shows that a MoS*_x_* nanophase with a high sulphur concentration (*х* ≥ 3) formed in the structure of these coatings. Such coatings are characterized by an XRD pattern with two strongly broadened bands at 2θ~15° and 2θ~40° [35,43].

**Figure 5:**
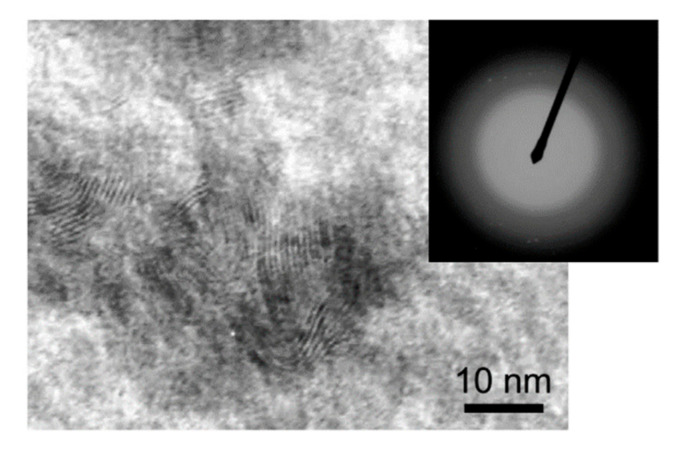
High-resolution TEM image of the Mo-S-C-H thin film obtained by reactive PLD at an H_2_S pressure of 5.5 Pa.

HRTEM studies of the Mo–S–C–H thin films confirmed their amorphous structure. Only in the Mo–S–C–H_5.5 films obtained at the lowest H_2_S, pressure, MoS_2_ nanocrystallites with laminar packing of atomic planes were found in some local regions. The size of these crystallites did not exceed 10 nm, and they were surrounded by an amorphous matrix. The concentration of MoS_2_ nanocrystallites in the Mo–S–C–H_5.5 film was not high, and their structure probably had a turbostratic character with a high degree of disordered local atomic packing. This was confirmed by the SAED pattern, consisting only of diffusely broadened rings (Figure 5), as well as by the results of the Raman studies of Mo–S–C–H films.

**Figure 6:**
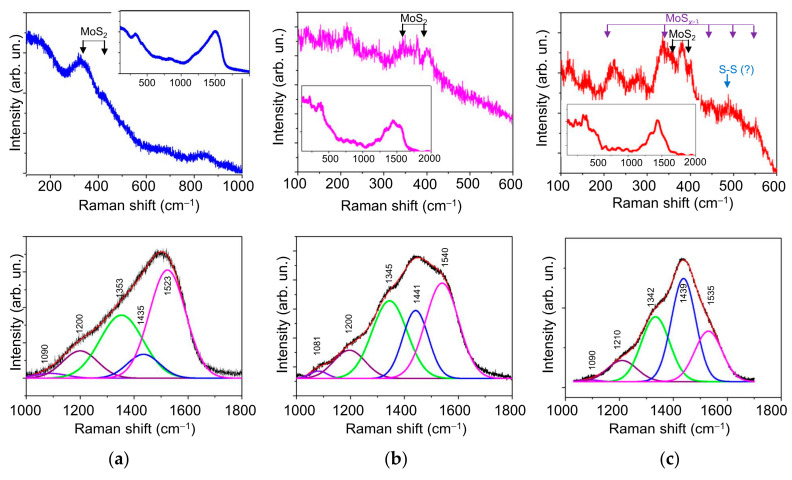
Raman spectra for the Mo–S–C–H thin-film coatings obtained by reactive PLD in the H_2_S gas at pressures of (**a**) 5.5, (**b**) 9, and (**c**) 18 Pa. The regions of Raman shifts corresponding to resonance light scattering by MoS*_x_*- and a-C(S,H)-based nanophases are shown at the top and bottom, respectively. The model of spectrum decomposition into the indicated peaks is discussed in the text. Inserts show the Raman spectra in the region from 100 to 2000 cm^−1^ that allows a correct comparison of the peak intensities for different nanophases.

The Raman spectrum for the Mo–S–C–H_9 coating in the frequency range of 100–600 cm^−1^ was in many respects similar to the spectrum of the Mo–S–C–H_5.5 coating (Figure 6). Broad peaks at 350 and 402 cm^−1^ indicated the formation of the MoS_2_ nanophase with a disordered atomic packing. The appearance in the spectrum of the Mo–S–C–H_9 coating of weak-intensity and broad peaks at ~200 and ~500 cm^−1^ suggested that, along with the MoS*_x_* nanophase, Mo_3_–S clusters could form. When such clusters are combined into a polymer-like network, MoS*_x_* compounds are formed, in which *х* ≥ 3 (Mo_3_S_12_/Mo_3_S_13_-type). The composition of Mo_3_-S clusters includes three Mo atoms connected in the Mo_3_–S triangle through monomers and/or dimers of S atoms (S^2−^/S_2_^2−^). With a sufficiently ordered packing of atoms in such clusters, narrow peaks are observed in the indicated frequency range; they correspond to various sulphur ligands [35,43,44,45]. In the Raman spectrum for the Mo–S–C–H_18 coating, the Mo_3_–S clusters corresponded to peaks at the following vibration modes: ν(Mo-Mo) at ~210 cm^−1^, ν(Mo-S)_coupled_ at ~330 cm^−1^, ν(Mo-S_apical_) at ~450 cm^−1^, ν(S-S)_terminal_ at ~520 cm^−1^, and ν(S-S)_bridging_ at 550 cm^−1^. In addition to these peaks, the spectrum of this coating exhibited peaks at 360, 380, and 401 cm^−1^, which could be due to atomic vibrations in the defective MoS_2_ nanophase.

When choosing a model for the decomposition of the Raman spectra for C-based nanophase in Mo–S–C–H films, we took into account the changes in the Raman spectra for a-C(S,H) films with increasing hydrogen sulphide pressure. The Raman spectra for a-C(S,H) films are shown in Figure S3 (Supplementary Materials). Figure S3 shows that, as the H_2_S pressure grows, i.e., with an increase in the concentration of sulphur and hydrogen in a-C(S,H) films, the contribution to the Raman spectra of the two peaks at frequencies of ~1220 cm^−1^ and ~1440 cm^−1^ rises as well. The intensity of these peaks in films having the highest concentration of S exceeded the intensity of the D (at ~1340 cm^−1^) and G (at ~1530 cm^−1^) peaks characteristic of pure a-C films. In this case, with an increase in the sulphur concentration, the *I*_D_/*I*_G_ ratio grew, which indicated an increase in the disordering (defectiveness) of atomic packing in graphite clusters.

Comparative analysis of the Raman spectra for the C-based nanophase in Mo–S–C–H and a-C (S,H) films showed (Figure 6) that the addition of Mo atoms to the depositing flux did not cause significant changes in the Raman spectra for the C-based nanophase. This was confirmed by the fact that the spectra of Mo–S–C–H coatings and a-C (S, H) films in the frequency range of 1000–1800 cm^−1^ were similar in many respects. An increase in the H_2_S pressure during RPLD of the Mo–S–C–H coatings led to an increase in the contribution to the spectrum of lines at ~1220 and ~1440 cm^−1^. Our analysis of the published data on Raman studies of Mo–S–C films formed by codeposition under magnetron sputtering (including the reactive one in an Ar/CH_4_ mixture) showed that the spectra of these films did not have properties characteristic of the spectra of the Mo–S–C–H films produced by RPLD. In the spectra of Mo–S–C films for the C-based nanophase, the positions of the D and G peaks, as well as the ratio of their intensity, tended to change, but new peaks did not appear [14,15,38,39,46,47]. Changes in the Raman spectra were caused by the influence of the MoS_2_ nanophase on the sp^2^/sp^3^ ratio (graphitization) and the level of mechanical stresses in the a-C(H) nanophase. More significant changes in the Raman spectrum of the carbon component in the a-C(S,H) films were found when using chemical vapour deposition in H_2_S, as well as during magnetron sputtering and pulsed laser ablation of composite targets made of a mixture of powders (MoS_2_, sulphur, graphite) [48,49,50]. Unfortunately, these works do not contain a sufficiently detailed analysis of the Raman spectra. Therefore, to investigate the C-based nanophase in the a-C(S,H) and Mo–S–C–H films obtained by RPLD, we used the approach proposed by Takeuchi et al. [51] for organic carbon sulphur materials.

Takeuchi et al. [51] have identified a class of organic carbon sulphur materials, the Raman spectrum of which has peaks at ~1250, 1350, 1440, and 1590 cm^−1^. The position of each peak has a tolerance of ±50 cm^−1^. The structure of such materials depends on the ratio of the intensities of these peaks. If the peak at 1400 cm^−1^ is the most intense, there is a large amount of the sp^3^ component of the G-band, and the majority of the carbon component form an undeveloped graphene (C-C) skeleton. Other peaks correspond to the sp^3^ component of the D band (~1250 cm^−1^), the sp^2^ component of the D band (~1350 cm^−1^), and the sp^2^ component of the G band (~1590 cm^−1^). The S‒S bond stretching vibration should peak at ~480 cm^−1^. This peak is present in the Raman spectrum of the Mo–S–C–H_18 coating (Figure 6c). The low intensity of this peak shows that RPLD was not effective for the formation of sulphur clusters. The process of dispersing sulphur in the carbon nanophase turned out to be more productive and caused a change in the local packing of carbon atoms and in the structure of the carbon skeleton. With a rise in the H_2_S pressure, i.e., with an increase in the concentration of sulphur, the contribution from the sp^3^ states caused by the introduction of sulphur in the structure of the carbon skeleton grew. At the same time, an increase in the intensity of the *I*_D_ peak at 1350 cm^−1^ (compared to *I*_G_ at 1540 cm^−1^) was indicative of a growing number of defects in the atomic packing of pure graphite clusters. Low intensity peak at 1080 cm^−1^ should be introduced for better fitting of the Raman spectrum.

**Figure 7:**
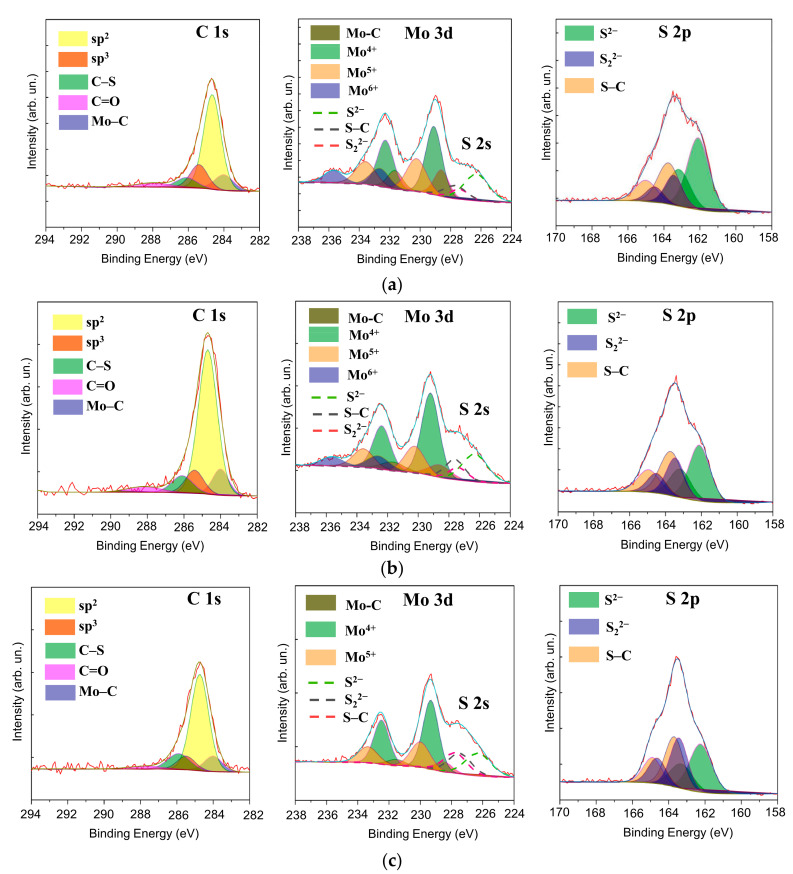
XPS spectra of C 1s, Mo 3d, and S 2p which were measured on the surface of the Mo–S–C–H thin-film coatings obtained by reactive PLD at H_2_S pressures of (**a**) 5.5, (**b**) 9, and (**c**) 18 Pa. For Mo 3d spectra, the S 2s spectra of different S species are indicated.

The RBS technique made it possible to determine the concentration of sulphur in the nanocomposite Mo–S–C–H coatings. This technique nevertheless does not distinguish between the sulphur content in MoS*_x_* and a-C(S,H) nanophases. Therefore, XPS measurements were carried out. Figure 7 shows the XPS spectra, revealing chemical bonds in the surface layer of the Mo-S-C-H coatings formed by RPLD at various H_2_S pressures. Decomposition of the Mo 3d spectrum showed that the chemical state of Mo atoms did not undergo significant changes with the increasing pressure of H_2_S. The Mo 3d spectra contained Mo3d_5/2_‒Mo3d_/2_ doublets, corresponding to Mo^2+^, Mo^4+^, Mo^5+^, and Mo^6+^. The electron binding energies for the Mo3d_5/2_ peaks at such valences of molybdenum were 228.6, 229.2, 230.3, and 232.6 eV, respectively. The dominance of the Mo^4+^ doublet indicated the effective formation of MoS_2_ and/or MoS*_x_* compounds (with packing Mo_3_‒C), in which *х* ≥ 3 [35,43,52,53]. The Mo^5+^ doublet may have been a result of the binding in the MoS_3_ compound with a linear packing of atoms into Mo‒S_3_ clusters [35,53,54]. The presence of a Mo^6+^ doublet with a low relative peak intensity indicated weak surface oxidation and the formation of Mo–O compounds [35,55]. With increasing H_2_S pressure, the intensity of the Mo^6+^ doublet weakened even more due to the chemical properties of hydrogen sulphide, which is a strong reducing agent. An increase in the H_2_S pressure caused a decrease in the contribution of the Mo^2+^ doublet, which corresponded to the Mo–C (Mo_2_C) bonds [55,56]. This was due to both an increase in the total sulphur concentration in the Mo–S–C–H films with a rise in the H_2_S pressure and, probably, due to an increased chemical activity of radicals formed upon activation of H_2_S by a laser plasma, compared with carbon atoms in a laser plasma from a graphite target. Considering the small contribution of the Mo‒C states, the effect of the carbide nanophase on the properties of M–S–C–H coatings was not considered in this work.

In the decomposition of the C 1s spectra for Mo–S–C–H coatings, it was assumed that C atoms could form chemical bonds with each other (C=C binding energy 284.6 eV and C‒C binding energy 285.5 eV), with S atoms (C‒S energy bonds 286.5 eV), and Mo atoms (C‒Mo binding energy 283.6 ÷ 284.2 eV) [36,37,56,57]. The peak with the highest binding energy (~289 eV) is usually attributed to C‒O bonds [57]. The analysis of the C 1s spectra showed that, at all H_2_S pressures, the peak corresponding to the sp^2^ bonds of C atoms dominated. As the H_2_S pressure grew, the contribution from the peak corresponding to С‒S bonds increased too. In this case, the contribution of the peak at 285.5 eV, corresponding to sp^3^ bonds of carbon atoms, slightly decreased. A thin film of organic contaminants containing CH*_x_* molecules may form on the surface of Mo–S–C–H coatings after being blown out of the PLD chamber. The presence of this film could have a definite effect on the results of studying the chemical state of carbon in Mo–S–C–H coatings, first of all, it could have increase the intensity of the XPS peak binding energy of ~284.5 eV.

The deconvolution of the S 2p spectra for Mo–S–C–H coatings allowed us to assume that S atoms can form chemical bonds with Mo atoms, which are characteristic of MoS*_x_* compounds with different values of *x*, as well as of chemical bonds with C atoms. The most common approach to analysing the chemical state of S atoms in molybdenum sulphides is the separation of two doublets S 2p_3/2_‒S 2p_1/2_ having “low” and “high” binding energies. A doublet with a low binding energy (the binding energy of the S 2p_3/2_ peak does not exceed 162.3 eV, and the S 2p_1/2_—163.5 eV) usually corresponds to the S^2−^ species characteristic of the MoS*_x_* compound [35,58]. A doublet with a high binding energy (the binding energy of the S 2p_3/2_ peak is ~162.8 ÷ 163.4.4 eV) corresponds to the S_2_^2^-species, which are characteristic of MoS*_x_* compounds, where clusters of Mo‒S_3_ and/or Mo_3_‒S are formed due to a high concentration of sulphur (*х* > 2), [35,43,52,53]. S atoms in the chemical bond with C atoms (‒S‒C‒S‒) correspond to the doublet S S 2p_3/2_‒S 2p_1/2_, in which the binding energy of the S 2p_3/2_ peak is 163.6 eV, and the S 2p_1/2_ peak equals 165.2 eV [57]. The choice of a model for describing the chemical state of S in a carbon matrix seems to be quite problematic since the binding energies of S atoms can strongly depend on the configuration of the nearest atoms. Thus, for a certain configuration of chemical bonds, the spin-orbit splitting of the S 2p state does not occur. Our analysis of the published results of XPS studies of sulphur-doped carbon materials has shown that even in the absence of spin-orbit splitting, a band at ~163.5 eV can dominate in the XPS spectra of S 2p (for example, a configuration of the C‒S_1÷2_‒C type), together with which a band at 165.0 eV (for instance, a configuration of the ‒C=S‒type) appears [59,60].

The application of the chosen model of the decomposition of the S 2p spectra showed that an increase in the H_2_S pressure resulted in a decrease in the concentration of S^2−^ states, and the contribution to the S_2_^2−^ states increased. The contribution of the species corresponding to the C‒S bonds increased as well. The calculation of the ratio *x* = S/Mo, taking into account S species (S^2−^ + S_2_^2−^) associated with Mo, and Mo species (Mo^4+^ + Mo^5+^) associated with S, showed that it was approximately 1.8, 2.5, and 4.0 for the Mo–S–C–H coatings obtained at pressures of 5.5, 9, and 18 Pa respectively. The composition of the C component in these coatings was described by the approximate formulas С_0.78_S_0.08_H_0.14_, C_0.73_S_0.11_H_0.16_, and C_0.62_S_0.18_H_0.2_. We assumed that H atoms are concentrated mainly in the a-C(S,H) nanophase. The calculated composition of the a-C(S,H) nanophase for Mo–S–C–H composite films differed from the composition of a-C(S,H) coatings obtained by RPLD at similar H_2_S pressures. The concentration of S atoms in the nanophase was lower than in the monophase thin-film coating. This could be attributed to the fact that S atoms deposited on the surface of the growing layer from the gas phase during ablation of a graphite target can be captured during the formation of the MoS*_х_* nanophase in the course of the subsequent ablation of the Mo target. Our calculations have shown that, as a result of the codeposition of Mo and C in reactive gas, the ratio *х* = S/Mo exceeds the ratio obtained earlier for MoS*_x_* films produced by RPLD of molybdenum in H_2_S at the same gas pressures.

### 3.3. Tribological Properties of Mo–S–C–H Films Obtained by RPLD

**Figure 8:**
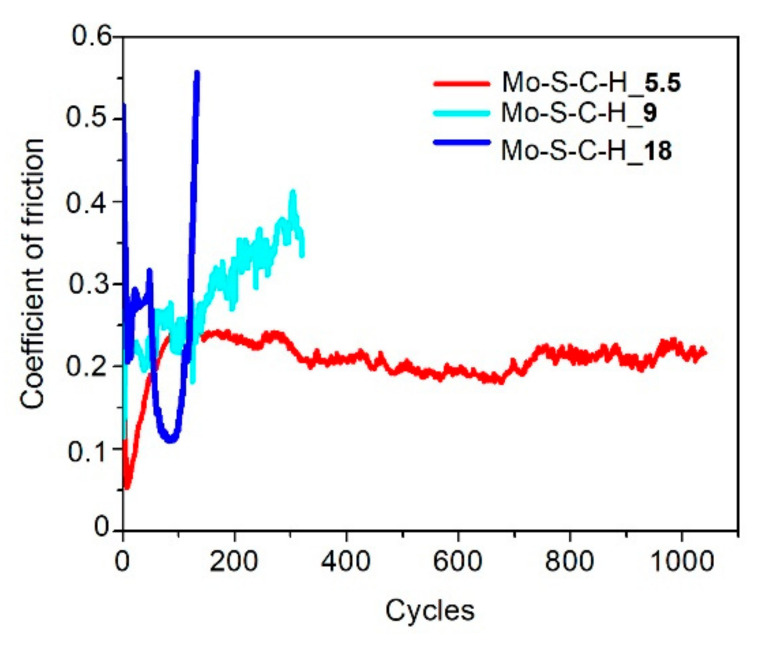
Characteristic evolution of the friction coefficient as a function of the cycle number for the Mo–S–C–H thin-film coatings obtained by reactive PLD at the pressures of H_2_S gas of 5.5, 9 and 18 Pa. Pin-on-disk tribometer testing was conducted in wet friction conditions (RH ~58%) at 22 °C.

**Figure 9:**
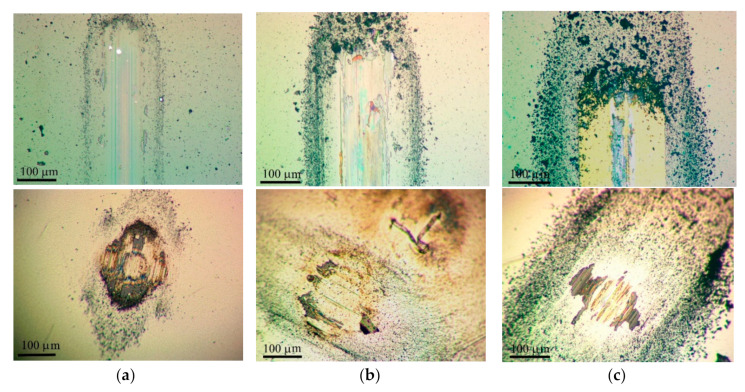
Optical images of wear tracks and wear scars formed on the steel substrates and steel balls for the Mo–S–C–H thin-film coatings obtained by reactive PLD at the different pressures of H_2_S gas: (**a**) 5.5, (**b**) 9, and (**c**) 18 Pa. Pin-on-disk tribometer testing was conducted in wet friction conditions (RH ~58%) at 22 °C. The test durations are indicated in Figure 8.

Figure 8 shows the results of measuring the average coefficient of friction as a function of the sliding cycle number of the steel counterbody over the Mo–C–S–H coatings in humid air. The Mo–C–S–H_5.5 coating obtained at the lowest H_2_S pressure turned out to be the most wear-resistant. The endurance of this coating exceeded 10^3^ cycles. For other coatings, the wear resistance did not exceed 200 cycles. The analysis of the wear tracks and the wear scar showed (Figure 9) that the low wear resistance of the Mo–C–S–H_9 and Mo–C–S–H_18 coatings is mainly due to the weak adhesion of the coatings to the substrate. The sliding of the counterbody caused the cracking of these coatings accompanied by the separation of microplates. Microplates accumulated around the track and adhered to the counterbody as well (Figure 9b,c).

The minimum value of the friction coefficient for the Mo–S–C–H_5.5 coating was 0.08; it was achieved after 10 sliding cycles. After 100 cycles, the coefficient of friction rapidly increased to 0.22 (±0.05), and this value remained constant throughout the entire testing period. A profilometric study of the wear crater showed that the wear rate of Mo–S–C–H_5.5, when tested in a humid atmosphere, was ~9 × 10^−7^ mm^3^/N m.

**Figure 10:**
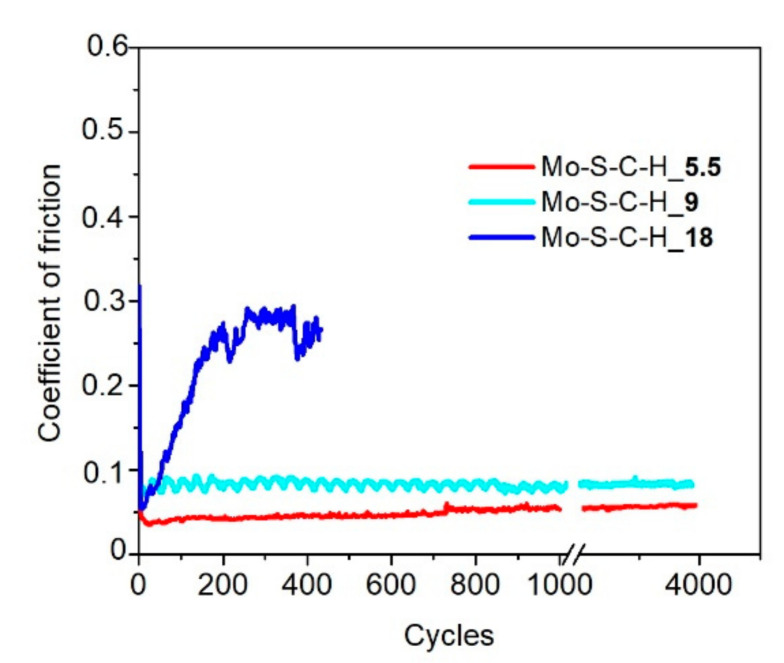
Characteristic evolution of the friction coefficient as a function of the cycle number for the Mo‒S‒C‒H thin-film coatings obtained by reactive PLD at the pressures of H_2_S gas of 5.5, 9, and 18 Pa. Pin-on-disk tribometer testing was conducted in dry friction conditions (air + Ar mixture, RH ~8%) at 22 °C.

**Figure 11:**
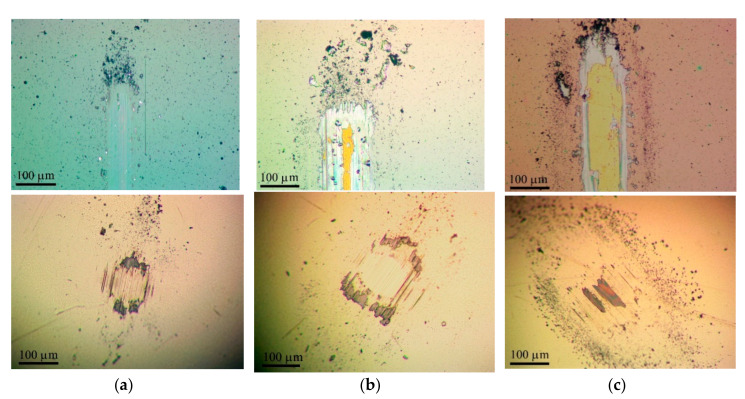
Optical images of wear tracks and wear scars formed on the steel substrates and steel balls for the Mo‒S‒C‒H thin-film coatings obtained by reactive PLD at the different pressures of H_2_S gas: (**a**) 5.5, (**b**) 9, and (**c**) 18 Pa. Pin-on-disk tribometer testing was conducted in dry friction conditions (air + Ar mixture, RH ~8%) at 22 °C. The test durations are indicated in Figure 10.

The Mo–S–C–H_5.5 coating showed better tribological properties compared to Mo–S–C–H_9 and Mo–S–C–H_18 coatings when tested in a dry atmosphere at room and low temperatures. Figure 10 demonstrates that, in dry friction conditions at 22 °C, the average coefficient of friction after the running-in period gradually increased from 0.03 (±0.05) to 0.05 (±0.05) with an increase in the test duration from 10 to 4 × 10^3^ cycles. The coating showed good adhesion to the substrate (Figure 11a). The wear rate of this coating was ~3 × 10^−7^ mm^3^/N m. The Mo–C–S–H_9 coating also had fairly good antifriction properties and durability despite its poor adhesion to the substrate. For this coating, the average coefficient of friction did not exceed 0.08 during the entire testing period (i.e., 4 × 10^3^ cycles). Weak adhesion of the coating to the substrate manifested itself in the formation of coating delamination areas in the track area. Coating separation caused the formation of micro-scales, which accumulated at the edges of the track (Figure 11b). The Mo–S–C–H_18 coating had poor tribological properties: it began to deteriorate immediately after the testing had started because of its weak adhesion to the substrate. Microscopic analysis showed that the loose fragments of the coating effectively adhered to the counterbody (Figure 11c).

**Figure 12:**
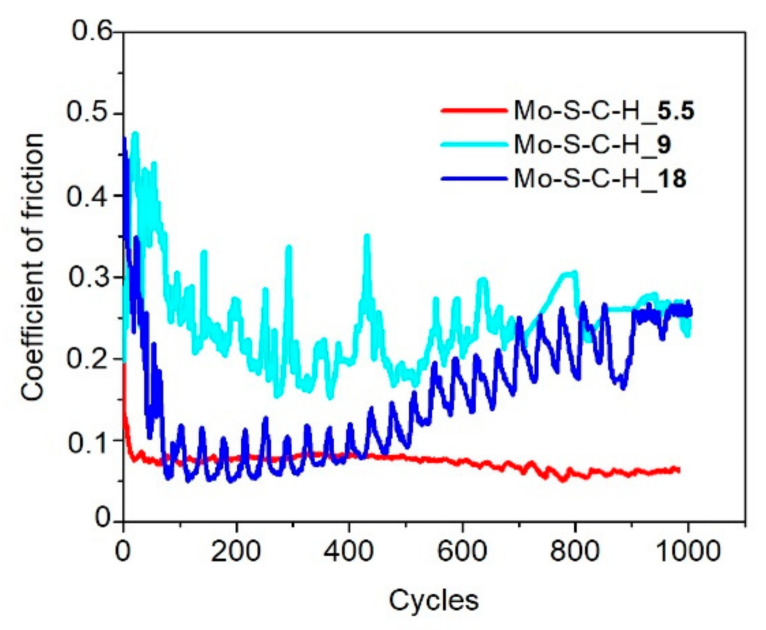
Characteristic evolution of the friction coefficient as a function of the cycle number for the Mo‒S‒C‒H thin-film coatings obtained by reactive PLD at the pressures of H_2_S gas of 5.5, 9, and 18 Pa. Pin-on-disk tribometer testing was conducted in dry friction conditions at −100 °C.

**Figure 13:**
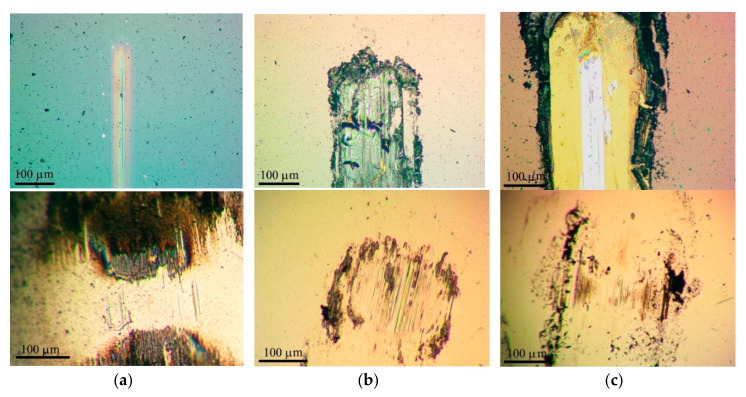
Optical images of wear tracks and wear scars formed on the steel substrates and steel balls for the Mo‒S‒C‒H thin-film coatings obtained by reactive PLD at the different pressures of H_2_S gas: (**a**) 5.5, (**b**) 9, and (**c**) 18 Pa. Pin-on-disk tribometer testing was conducted in dry friction conditions at −100 °C. The test durations are indicated in Figure 12.

During testing in dry friction conditions at −100 °C, the average coefficient of friction for the Mo–S–C–H_5.5 coating did not exceed 0.08 (±0.1) over 10^3^ sliding cycles (Figure 12). A shallow track formed on the coating surface, and the wear rate of the coating did not exceed 1.6 × 10^−7^ mm^3^/N m (Figure 13a). The sliding of the counterbody over the Mo–C–S–H_9 and Mo-C-S-H_18 coatings was accompanied by noticeable changes in the average coefficient of friction in the range from 0.05 to 0.25 (Figure 12). In this case, the coatings retained their continuity, but they could deform and crack (Figure 13b,c). Sliding of the ball on the Mo–C–S–H_9 and Mo–C–S–H_18 coatings caused more intensive wear of the counterbody than sliding on the Mo–S–C–H_5.5 coating.

## 4. Discussion

Our comparison of the tribological properties of the Mo–S–C–H coatings obtained at different pressures of hydrogen sulphide showed that an increase in pressure negatively affected both the average coefficient of friction and the wear resistance of the coatings under various tribological testing conditions. To explain this result, it is necessary to assess the possible effect of the nanophases of these coatings on the tribological properties. A change in the conditions of RPLD caused significant changes in both the MoS*_x_* and C-based nanophase. An increase in the H_2_S pressure caused a rise in the *x* = S/Mo ratio from ~1.8 to ~4.0. Fominski et al. [35] found that increasing *x* to 4 can significantly worsen the tribological properties of MoS*_x_* coatings. For this reason, the generally unsatisfactory properties of the Mo–S–C–H_18 coatings could be caused by inclusions of the MoS_4_ nanophase. At 2 ≤ *х* ≤ 3, the properties of MoS*_x_* coatings depend on the conditions of tribological tests. In a humid atmosphere, after 400 cycles of sliding, MoS_2_ and MoS_3_ monophase coatings had the friction coefficient of ~0.2 and ~0.12, respectively. Additional studies of MoS*_x_* thin film coatings formed by the RPLD at H_2_S gas pressure of 5.5 Pa revealed unsatisfactory tribological properties. Under various tribotest conditions, these coatings began to break down almost immediately after the start of the counterbody sliding (Supplementary Materials, Figure S4). The Mo–S–C–H_9 coating, containing MoS*_х_*_~2.5_ nanophase, did not show good antifriction properties in a humid atmosphere, which indicates the possibility of a negative effect of another nanophase, which is part of this coating—the a-C(S,H) component formed at H_2_S pressure of 9 Pa. The antifriction properties of the Mo–S–C-H_5.5 coating containing the MoS_1.8_ nanophase correlated rather well with the properties of a single-layer MoS_2_ coating.

For comparing the tribological properties of the Mo–S–C–H coatings produced by RPLD with those of Mo–S–C coatings obtained by more traditional deposition methods (mainly, magnetron sputtering), it is necessary to take into account a number of important factors, such as the composition of the coatings, air humidity and the load applied on the counterbody [15,61]. In the friction tests of the Mo–S–C–H_9 and Mo–S–C–H_18 coatings in a humid atmosphere with an increased load on the ball (5 N), the average friction coefficient was ~0.066, and the endurance did not change with an increase in the load and was 100 cycles. The friction coefficient for the MoS_2_ and Mo–S–C coatings at an increased carbon concentration (≥30 at.%) in a humid atmosphere (RH ≥ 50%) varied from ~0.1 to 0.3, and the wear rate varied from ~5 × 10^−7^ mm^3^/N m to 20 × 10^−7^ mm^3^/N m. The best performance is achieved only by alloying these coatings with metals (Ti, Pb, and others) [62,63,64]. Thus, it can be assumed that the Mo–S–C–H_5.5 nanocomposite coating, when tested in a humid atmosphere (RH ≥ 50%), is inferior in its tribological properties only to the best samples of the MoS_2_ and Mo–S–C coatings doped with metals.

During friction testing in dry friction conditions at room temperature, the coefficient of friction for the MoS_2_ and MoS_3_ coatings was 0.08 and 0.1 respectively after 400 cycles of sliding [35]. To compare, the Mo–S–C–H_9 coating containing the MoS*_х_*_~2.5_ nanophase also provided a fairly stable and low coefficient of friction (~0.08). However, the Mo–S–C–H_5.5 coating containing the MoS_1.8_ nanophase provided a more effective decrease in the friction coefficient (down to 0.03), which was probably due to the positive effect of the a-C(S,H) component formed at an H_2_S pressure of 5.5 Pa. The friction coefficient value of ~0.05 has been noted in many studies of pure TMD and nanocomposite TMD+C coatings when tested under dry friction conditions (~5% ≤ RH ≤ ~30%) at moderate loads on the counterbody (for example, [15,47,61,65,66,67]). The wear rate can be reduced to ~2 × 10^−7^ mm^3^/N m, which is achieved by alloying with metals. Fundamentally lower values of the friction coefficient (~0.005 ÷ 0.01) during sliding in dry friction conditions are achieved by creating nanoscale layers of MoS_2_ and a-C(H) [17,68]. The friction coefficient for the Mo–S–C–H_5.5 coating equal to 0.03 at a humidity of RH ~8% turned out to be slightly lower than the values for the MoS*_x_* and Mo–S–C coatings formed by magnetron sputtering. Yet this coating was clearly inferior to the MoS_2_/а-С(Н) nanolayer coatings exhibiting superlubricity properties. This could be caused by both the suboptimal composition of the coating nanocomponents and by the fact that the a-C(S,H) nanophase formed during RPLD did not provide ultralow friction in combination with the MoS_1.8_ nanophase.

For the MoS_2_ and MoS_3_ monophase coatings, the average coefficient of friction under extreme test conditions (−100 °C) was 0.18 and 0.08 respectively after 400 cycles of sliding [35]. The Mo–S–C–H_9 coating containing the MoS*_х_*_~2.5_ nanophase provided sliding with a higher coefficient of friction (0.2–0.3). A lower and stable coefficient of friction (~0.08) was determined for the Mo–S–C–H_5.5 coating containing the MoS_1.8_ nanophase. At this stage, it is difficult to do a comparative analysis of the tribological properties of Mo–S–C–H coatings obtained by RPLD and coatings of the same type obtained by other techniques since there is no information on tribotests of various coatings at −100 °C in the literature. A comparison of the tribological properties of the monophase MoS*_x_* and nanocomposite Mo–S–C–H coatings indicates a significant effect of the a-C(S,H) phase on the tribological properties of the Mo–S–C–H nanocomposite coatings under friction at low temperatures.

A comparative analysis of the tribological properties of the Mo–S–C–H nanocomposite coatings obtained by RPLD with the properties of MoS*_x_* coatings (also obtained by RPLD) and TMD+C coatings prepared by more traditional techniques showed the importance of collecting additional information on the tribological properties of a-C (S,H) coatings formed by RPLD. A review of the literature shows that sulphur can significantly change the tribological and mechanical properties of a-C(S,H) coatings, and the effect of the introduction of sulphur depends on its concentration and the concentration of hydrogen to a considerable degree [69,70,71]. The tribological properties of a-C(S,H) coatings formed by RPLD require further research. Our results of tribological studies of a-C(S,H) thin-film coatings obtained by RPLD on steel substrates are presented in Supplementary Materials, Figures S6–S11.

It is important to note that pure a-C coatings prepared by the RPLD in a vacuum delaminated from the substrate one to two days after the sample had been taken out into the air from the deposition chamber. The SiC sublayer failed to provide sufficient adhesion of the a-C coating to the substrate, which can be explained by a high level of mechanical stress in the carbon film. Tribotests of a-C(S,H) thin-film coatings under various friction conditions showed that an increase in the concentration of sulphur due to an increase in H_2_S pressure had a negative effect on both antifriction properties and fracture resistance. Even if the MoS_2.5_ nanophase in the Mo-S-C_9 coating could provide good antifriction properties under certain conditions, the a-C(S,H)_9 phase, having an approximate composition of C_0.73_S_0.11_H_0.16_, would not let it happen. The degradation of the a-C(S,H)_9 and a-C(S,H)_18 coatings occurred by cracking and pilling off from the substrate. It can clearly be seen in the shape of the wear debris formed after friction test. These were mainly microplates accumulating at the edges of the track and adhering to the counterbody.

When under dry friction conditions, only the a-C(S,H)_5.5 coating demonstrated good antifriction properties. These coatings were produced from graphite by reactive PLD at an H_2_S pressure of 5.5 Pa. The friction coefficient did not exceed 0.03 during 10^3^ cycles of the sliding of the ball. In that case, the surface of the a-C(S,H)_5.5 coating underwent a slight wear, and the wear scar on the surface of the steel counterbody was just incipient. Obviously, the qualitative tribological properties of the Mo–S–C–H_5.5 nanocomposite coating under dry friction conditions were due to the influence of the a-C(S,H) phase. Under other friction conditions, the tribological properties of the Mo–S–C–H_5.5 coating depended on the synergistic effect of the formation of a composition of the MoS*_х_* and a-C(S,H) nanophases. Under friction in a humid atmosphere, the MoS*_х_*_~1.8_ + a-C(S,H) combination provided the coefficient of friction characteristic of both phases, but changed the wear mechanism of the a-C(S,H) phase, preventing its cracking and delamination from the substrate. At low temperatures (−100 °C), the synergy effect of the MoS*_х_*_~1.8_ and a-C(S,H) phases caused a rather low coefficient of friction and high wear resistance. The friction coefficient was found to be lower than the values typical for the MoS*_x_*_~2_ and a-C(S,H)_5.5 thin-film coatings.

**Figure 14:**
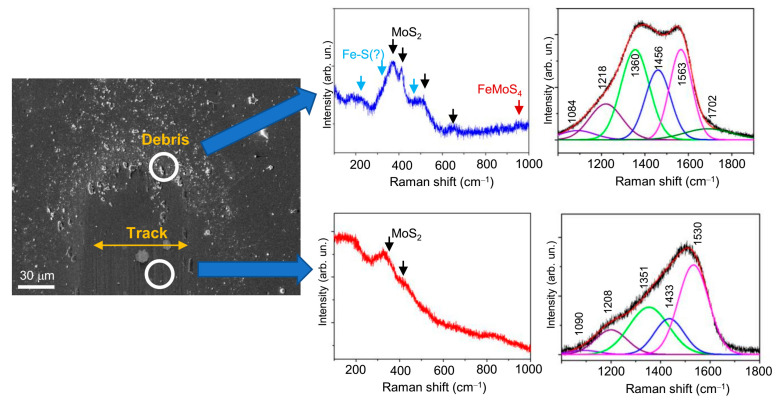
SEM image and Raman spectra for the Mo‒S‒C‒H_5.5 thin-film coating subjected to pin-on-disk test at 22 °C in wet friction conditions (RH ~58%).

**Figure 15:**
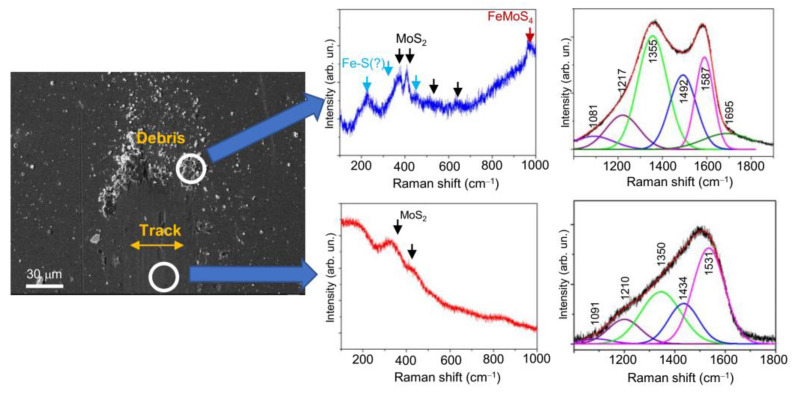
SEM image and Raman spectra for the Mo-S-C-H_5.5 thin-film coating subjected to pin-on-disk test at 22 °C in dry friction conditions (RH ~8%).

**Figure 16:**
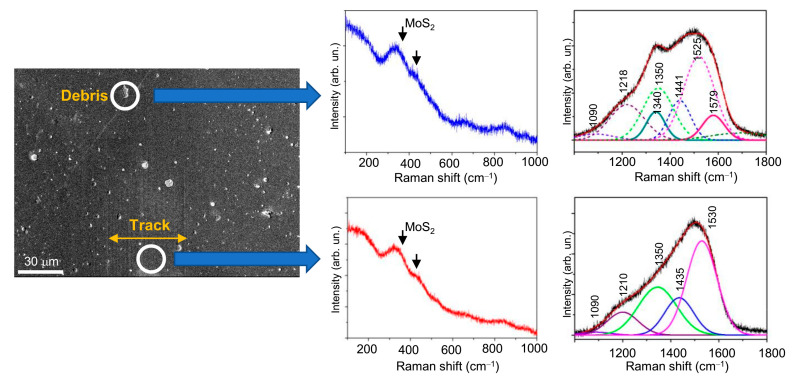
SEM image and Raman spectra for the Mo-S-C-H_5.5 thin-film coating subjected to pin-on-disk test in dry friction conditions at −100 °C.

The Raman studies of the wear track on the nanocomposite Mo–S–C–H_5.5 coating showed (Figure 14, Figure 15 and Figure 16) that sliding friction of steel counterbody caused subtle changes in the Raman spectra measured in a middle region of the track. This indicates that the triboinduced changes occurred in a very thin near-surface layer of the coating. These changes manifested in an increase in the contribution of the peak at 1433 cm^−1^. This could be due to the accumulation (an increase in the concentration) of S atoms in the surface layer of the coating during friction. No changes in the structure of the MoS*_x_* nanophase were found. Comparison of Raman peaks for the Mo–S–C–H_5.5 coating before (Figure 6a) and after the friction testing showed no noticeable changes in the spectra in the frequency range of 800–1000 cm^−1^. This indicated a high resistance of Mo–S–C–H_5.5 coatings to oxidation in a humid atmosphere.

МRS analysis of the wear debris accumulated near the counterbody reversal points showed (Figure 14 and Figure 15) that the chemical state of the wear debris, and hence the chemical state of the tribofilm, depended on the tribotest conditions. Under friction in a humid atmosphere, the wear debris contained the crystalline phase of MoS_2_. This was indicated by the appearance of rather narrow peaks at ~370 and 405 cm^−1^ caused by first-order reflection for the 2H-MoS_2_ phase. Formed during triboinduced crystallization, this phase can cause peaks at ~520 and 650 cm^−1^, which appear due to the second-order vibration modes of MoS_2_ [64]. Another peak at ~950 cm^−1^ may have occurred due to the formation of a Fe‒Mo‒O compound (for example, FeMoO_4_) as a result of the tribochemical reaction of the surface of the steel counterbody with the surface of the Mo–S–C–H_5.5 coating in the presence of adsorbed water molecules. The Raman spectrum of the wear debris contained peaks that could be attributed to the Fe‒S phase. The Raman spectrum of the Fe‒S nanoparticulate phase has the most intense peaks at ~215, 323, and 463 cm^−1^ [72]. Further research is needed to confirm the formation of the Fe‒S phase. Figure 14 demonstrates the peaks for the Fe‒S nanophase, but they are indicated by a question mark (?).

In addition to the MoS_2_, FeMoO_4_ and, possibly, Fe‒S phases, the wear debris contained a graphite-like phase, which corresponded to a doublet containing broadened G (at ~1563 ÷ 1587 cm^−1^) and G (at 1355 ÷ 1360 cm^−1^) peaks. The formation of such a C-based phase is typical of triboinduced changes in TMD+C coatings (see, for example, [15,24,46,73]). An important factor influencing friction is the local environment and atom packing in that phase. The Raman spectra for the wear particles formed on the Mo-SC-H_5.5 coating after friction in a humid and dry atmosphere, differ in both the position of the G peak and the width of the G and D peaks. Under friction in a dry atmosphere, these peaks turn out to be narrower, and the G peak shifts to higher frequencies up to 1587 cm^−1^. It shows that during friction in a humid atmosphere, graphitization of the surface layer of the coating was insufficient, and the tribofilm structure was highly disordered. Probably, this was due to the fact that, at increased air humidity, the contact of the coating with the counterbody was modified because of the adsorption of water molecules and slowed graphitization. This resulted a relatively high coefficient of friction (~0.2) for the Mo–S–C–H_5.5 coating in a humid atmosphere.

The analysis of published data on the triboinduced graphitization of the interface layer for TMD+C and a-C(H) coatings and their comparison with the results of this work showed that S atoms incorporated into the a-C(H) phase during the RPLD of Mo–S–C–H_5.5 films did not have a significant effect on the formation of tribofilms during friction in a dry atmosphere. The relatively narrow G and D peaks located at 1355 and 1580 cm^−1^ respectively, correspond to the graphite/graphene-like packing of atoms with a laminar structure [46,71,73,74,75]. The incorporation of S atoms into the graphene structure may not cause noticeable changes in the Raman spectrum of graphene [76]. Still, under certain conditions when graphene interacts with sulphur, a band may appear at ~1440 cm^–1^ in the graphene Raman spectrum [77], the nature of which was considered in the above analysis of the Raman spectra of Mo–S–C–H and a-C(S,H) films with a high sulphur concentration. During sliding friction against the Mo–S–C–H_5.5 coating in a dry atmosphere, along with the graphitization of the a-C(S,H) phase, crystallization of the MoS_2_ nanophase occurred; therefore, this phase could not compromise the positive antifriction properties of the a-C(S,H) phase.

During the tests of the Mo–S–C–H_5.5 coating at −100 °C, the formation of wear debris and their accumulation on the sample surface did not occur in an intensive way. This may be attributed to the fact that these particles were very small and effectively adhered to the counterbody surface (Figure 13a). When measuring the Raman spectrum on a single small particle, light was registered; it was resonantly scattered by both the particle and the coating (Figure 16). The main contribution to the spectrum from the particle was the appearance of weak peaks at ~1340 and 1579 cm^−1^, that indicated weak graphitization of the material in the wear debris. No signs of crystallization of the MoS*_x_* nanophase were found. It shows that that the mechanism of friction and wear of the Mo–S–C–H_5.5 coating at −100 °C differed from that for the MoS_2_ coatings, which underwent effective crystallization under similar test conditions [35]. This could be due to the fact that inclusions of the MoS*_x_* nanophase into the amorphous a-C(S,H) matrix could reduce the level of mechanical stresses in the coating. The nanocomposite Mo–S–C–H_5.5 coatings wear out by the mechanism of layer-by-layer removal of the surface layer while the a-C(S,H) coatings showed a tendency to cracking. The presence of the MoS*_x_* phase in the nanocomposite coating could have an effect on the adsorption of water molecules on the coating surface at low temperatures, and possibly on the formation of water microcrystals. The influence of these factors on the tribological properties of Mo–S–C–H coatings at low temperatures require a further in-depth study.

## 5. Conclusions

Pulsed laser deposition of Mo–S–C–H thin-film nanocomposite coatings from Mo and graphite targets in H_2_S reactive gas ensures effective saturation of the formed layers with S atoms. The penetration of S atoms causes the formation of the MoS*_x_* nanophase, but also significantly changes the chemical state of the a-C(S,H) phase. In this work, we chose specific RPLD conditions under which the ablation time of the Mo target was twice as long as the ablation time of the C target. With an increase in the H_2_S pressure from 5.5 to 18 Pa, the concentration of S in the MoS*_x_* nanophase increased from *x*~1.8 to *x*~4. Significant changes were observed in the chemical composition of the a-C(S,H) phase. At 5.5 Pa, the composition was described by the formula С_0.78_S_0.08_H_0.14_, and at 18 Pa, an increase in the concentration of sulphur caused the formation of C_0.62_S_0.18_H_0.2_. For the coatings obtained at 5.5 and 18 Pa, the ratio of the number of atoms in the MoS*_x_* and a-C(S,H) phases was ~40/60 and 60/40, respectively. At the lowest S concentration, the local packing of atoms in the MoS*_х_* nanophase was close to the laminar packing characteristic of the turbostratic MoS_2_ structure. In this case, the a-C(S,H) nanophase was amorphous with a predominance of sp^2^ bonds between C atoms. An increase in the concentration of S atoms caused the formation of MoS*_x_* clusters, in which the Mo_3_‒S packing began to dominate. In this case, the configuration of the local packing of atoms in the a-C(S,H) phase was significantly modified due to the efficient formation of C–S bonds.

The best tribological properties were found for the Mo–S–C–H_5.5 nanocomposite coatings obtained at an H_2_S pressure of 5.5 Pa. At higher H_2_S pressures, an increase in the concentration of S atoms both in the MoS*_x_* nanophase and in the a-C(S,H) nanophase caused a noticeable deterioration in the tribological properties of the Mo–S–C–H_9 and Mo–S–C–H_18 nanocomposite coatings. The tribological properties of the Mo–S–C–H_5.5 thin-film coatings were superior to those of the MoS_2_ coatings under various friction test conditions. However, in a humid atmosphere, the antifriction properties of the Mo–S–C–H_5.5 coating turned out to be worse than the properties of the MoS_3_ coating. MoS_2_ and MoS_3_ coatings were also obtained by RPLD [35]. The friction and wear of Mo–S–C–H_5.5 coatings in a humid atmosphere at 22 °C and in a dry atmosphere at −100 °C were due to the synergy effect of the MoS*_x_* and a-C(S,H) nanophases. Under dry friction conditions, the sufficiently high-quality tribological properties of Mo–S–C–H_5.5 resulted from the dominant influence of the a-C(S,H) phase, which is the most suitable for these conditions and has a relatively optimal chemical composition.

## Supplementary Materials

The following are available online at https://www.mdpi.com/2079-4991/10/12/2456/s1, Figure S1: Experimental and simulated RBS (left) and ESDA (right) spectra of the films prepared on Si substrates by PLD of carbon under vacuum conditions and in H_2_S gas with different pressures. The RBS spectrum of the carbon film deposited in vacuum contains (residual gas pressure was ~10^−3^ Pa) a peak at the channel number 200. This peak is due to scattering of ions by sulfur atoms that have been adsorbed on the surface of the Si substrate before the carbon film deposition, Figure S2: Typical SEM images (two magnifications) of a-C (S, H) film obtained on a steel substrate by reactive PLD in H_2_S, Figure S3: Raman spectra for (a) a-C(H) and (b‒d) a-C(S,H) films obtained by PLD on Si substrate (a) under vacuum conditions and in reactive H_2_S gas at pressures of (b) 5.5, (c) 9, and (d) 18 Pa. The model of spectrum decomposition into the indicated peaks is discussed in the text of the article, Figure S4: The friction force (in relation with the normal force) evolution during reciprocate sliding of the counterbody over the MoS*_x_* coating under different environmental conditions. The MoS*_x_* coating was obtained on the steel substrate by reactive PLD at H_2_S pressure of 5.5 Pa, Figure S5: Optical images of wear tracks formed on the steel substrates for MoS*_x_* thin-film coating obtained by reactive PLD at H_2_S pressure of 5.5 Pa. Pin-on-disk tribometer testing was conducted in (a) wet friction conditions (RH ~50%) at 22 °C, (b) dry friction conditions (RH ~8%) at 22 °C, and (c) dry friction conditions at −100 °C. The test durations are indicated in Figure S4, Figure S6: Characteristic evolution of the friction coefficient as a function of the cycle number for a-C(S,H) thin-film coatings obtained by reactive PLD at the pressures of H_2_S gas of 5.5, 9 and 18 Pa. Pin-on-disk tribometer testing was conducted in wet air (RH ~58%) at 22 °C, Figure S7: Optical images of wear tracks and wear scars formed on the steel substrates and steel balls for a-C(S,H) thin-film coatings obtained by reactive PLD at the different pressures of H_2_S gas: (a) 5.5, (b) 9, and (c) 18 Pa. Pin-on-disk tribometer testing was conducted in wet air (RH ~58%) at 22 °C. The test durations are indicated in Figure S6, Figure S8: Characteristic evolution of the friction coefficient as a function of the cycle number for a-C(S,H) thin film coatings obtained by reactive PLD at the pressures of H_2_S gas of 5.5, 9, and 18 Pa. Pin-on-disk tribometer testing was conducted in dry friction conditions (air+Ar mixture, RH ~8%) at 22 °C, Figure S9: Optical images of wear tracks and wear scars formed on the steel substrates and steel balls for a-C(S,H) thin film coatings obtained by reactive PLD at the different pressures of H_2_S gas: (a) 5.5, (b) 9, and (c) 18 Pa. Pin-on-disk tribometer testing was conducted in dry friction conditions at 22 °C. The test durations are indicated in Figure S8, Figure S10: Characteristic evolution of the friction coefficient as a function of the cycle number for a-C(S,H) thin-film coatings obtained by reactive PLD at the pressures of H_2_S gas of 5.5, 9 and 18 Pa. Pin-on-disk tribometer testing was conducted in dry friction conditions at −100 °C, Figure S11: Optical images of wear tracks and wear scars formed on the steel substrates and steel balls for a-C(S,H) thin-film coatings obtained by reactive PLD at the different pressures of H_2_S gas: (a) 5.5, (b) 9, and (c) 18 Pa. Pin-on-disk tribometer testing was conducted in dry friction conditions at −100 °C. The test durations are indicated in Figure S10.
