# Supplementary material for: Specific Features of Reactive Pulsed Laser Deposition of Solid Lubricating Nanocomposite Mo–S–C–H Thin-Film Coatings
Source: Nanomaterials (Basel). 2020 Dec 8;10(12):2456. doi: 10.3390/nano10122456 (PMC7764125; doi:10.3390/nano10122456)
Supplement: Supplementary file 1 [file nanomaterials-10-02456-s001.pdf]

nanomaterials

Supplementary Materials

Specific Features of Reactive Pulsed Laser Deposition of Solid Lubricating Nanocomposite Mo-S-C-H Thin-Film Coatings

Vyacheslav Fominski [1,*], Dmitry Fominski [1], Roman Romanov [1], Mariya Gritskevich [1], Maxim Demin [2], Petr Shvets [2], Ksenia Maksimova [2] and Alexander Goikhman [2]

1 National Research Nuclear University MEPhI (Moscow Engineering Physics Institute), Kashirskoe sh., 31, 115409 Moscow, Russia; dmitryfominski@gmail.com (D.F.); limpo2003@mail.ru (R.R.); mgritskevich@yandex.ru (M.G.)
2 Immanuel Kant Baltic Federal University, A. Nevskogo St 14, 236016 Kaliningrad, Russia; sterlad@mail.ru (M.D.); pshvets@kantiana.ru (P.S.); xmaksimova@gmail.com (K.M.); aygoikhman@gmail.com (A.G.)
* Correspondence: vyfominskij@mephi.ru

Pulsed laser ablation of graphite target under vacuum and in reactive H_2S gas

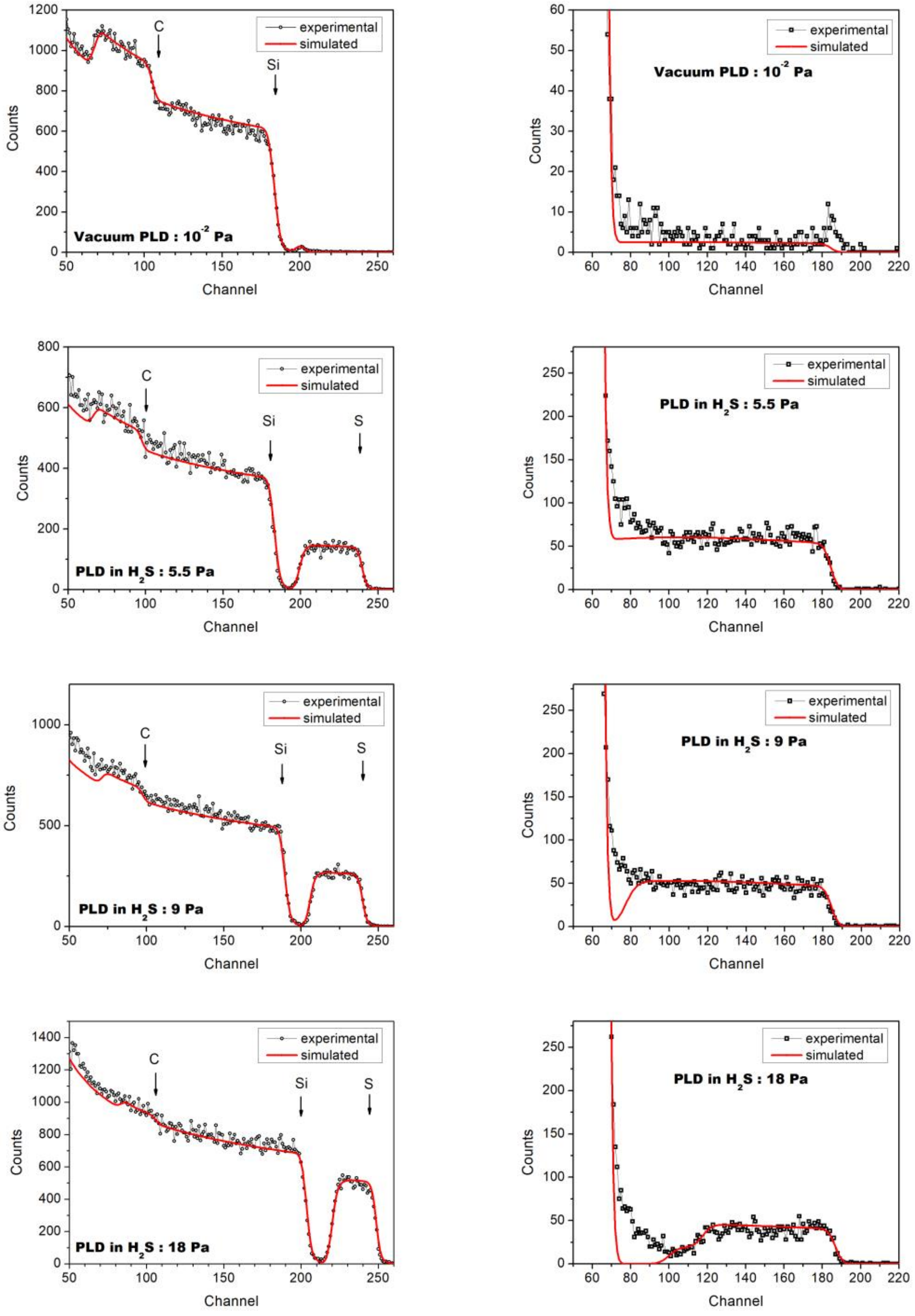

Figure S1. Experimental and simulated RBS (left) and ESDA (right) spectra of the films prepared on Si substrates by PLD of carbon under vacuum conditions and in H_2S gas with different pressures. The RBS spectrum of the carbon film deposited in vacuum contains (residual gas pressure was ~10^{-3} Pa) a peak at the channel number 200. This peak is due to scattering of ions by sulfur atoms that have been adsorbed on the surface of the Si substrate before the carbon film deposition.

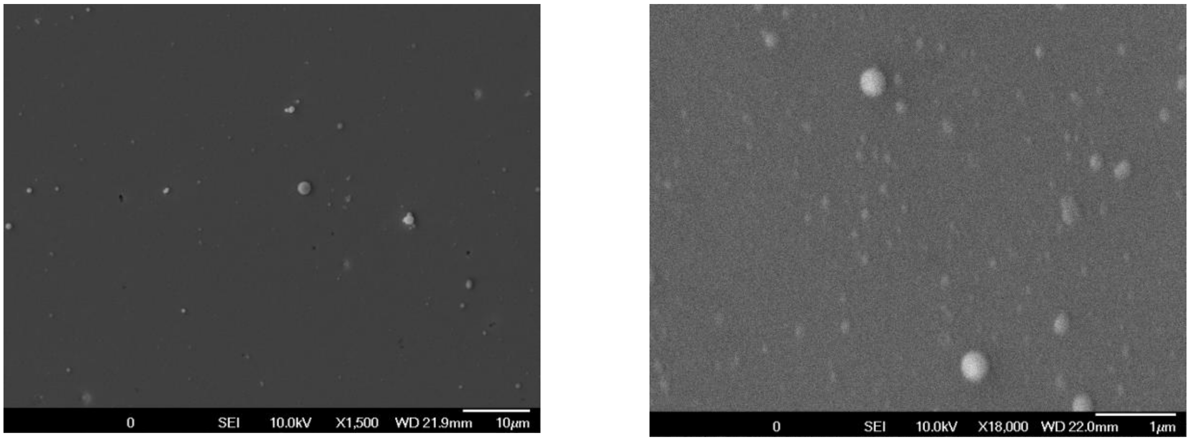

Figure S2. Typical SEM images (two magnifications) of a-C (S, H) film obtained on a steel substrate by reactive PLD in H_2S.

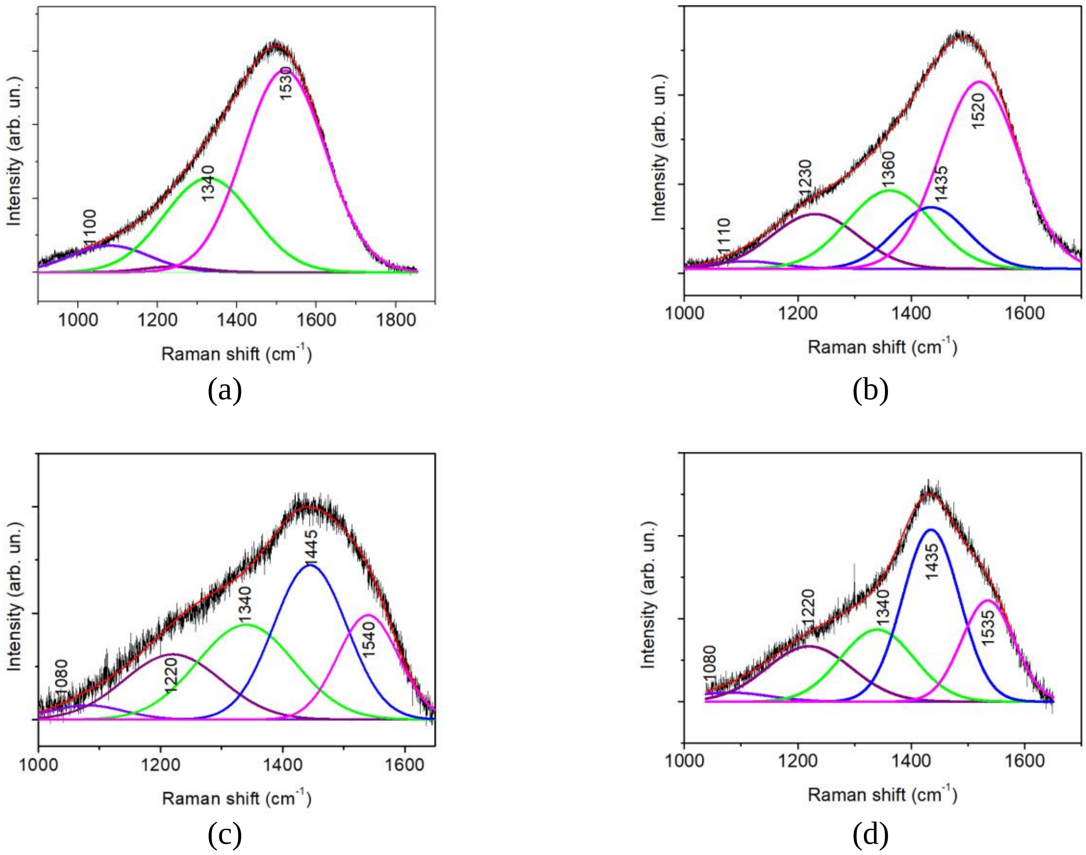

Figure S3. Raman spectra for (a) a-C(H) and (b–d) a-C(S,H) films obtained by PLD on Si substrate (a) under vacuum conditions and in reactive H_2S gas at pressures of (b) 5.5, (c) 9, and (d) 18 Pa. The model of spectrum decomposition into the indicated peaks is discussed in the text of the article.

Tribological properties of MS_x thin-film coating obtained by reactive PLD at H_2S pressure of 5.5 Pa

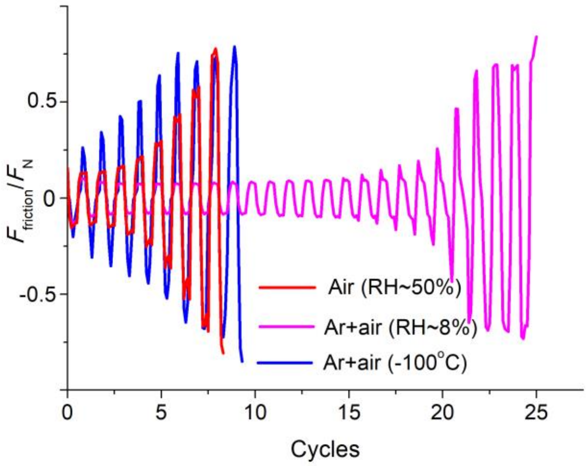

Figure S4. The friction force (in relation with the normal force) evolution during reciprocate sliding of the counterbody over the MoS_x coating under different environmental conditions. The MoS_x coating was obtained on the steel substrate by reactive PLD at H_2S pressure of 5.5 Pa.

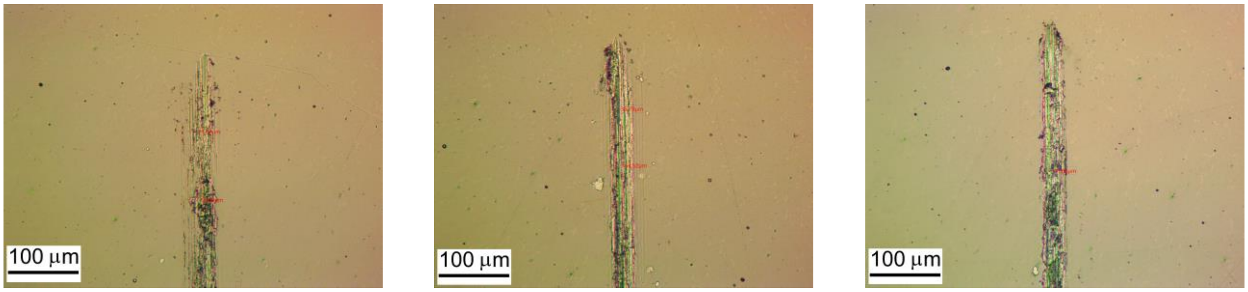

(a) (b) (c)

Figure S5. Optical images of wear tracks formed on the steel substrates for MoS_x thin-film coating obtained by reactive PLD at H_2S pressure of 5.5 Pa. Pin-on-disk tribometer testing was conducted in (**a**) wet friction conditions (RH~50%) at 22 °C, (**b**) dry friction conditions (RH~8%) at 22 °C, and (**c**) dry friction conditions at -100 °C. The test durations are indicated in Figure S4.

Tribological properties of a-C(S,H) thin film coatings obtained by reactive PLD

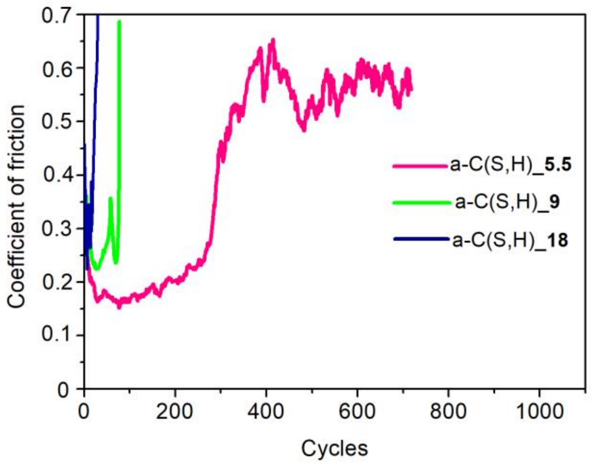

Figure S6. Characteristic evolution of the friction coefficient as a function of the cycle number for a-C(S,H) thin-film coatings obtained by reactive PLD at the pressures of H_2S gas of 5.5, 9 and 18 Pa. Pin-on-disk tribometer testing was conducted in wet air (RH~58%) at 22 °C.

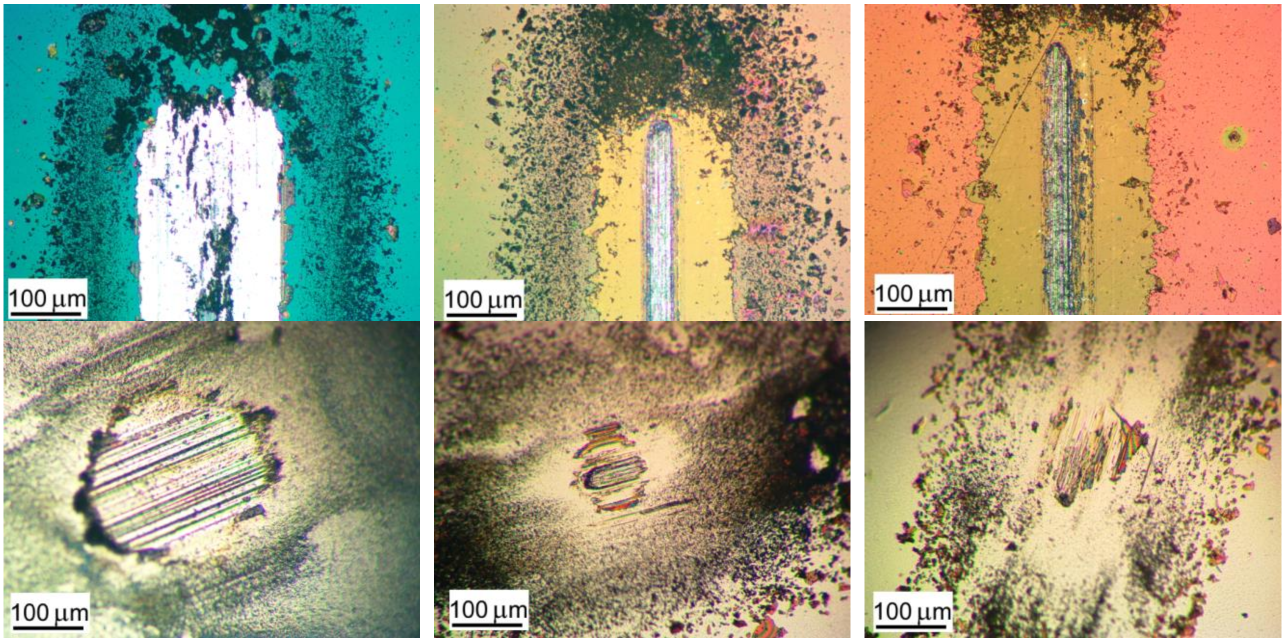

(a) (b) (c)

Figure S7. Optical images of wear tracks and wear scars formed on the steel substrates and steel balls for a-C(S,H) thin-film coatings obtained by reactive PLD at the different pressures of H_2S gas: (**a**) 5.5, (**b**) 9, and (**c**) 18 Pa. Pin-on-disk tribometer testing was conducted in wet air (RH~58%) at 22 °C. The test durations are indicated in Figure S6.

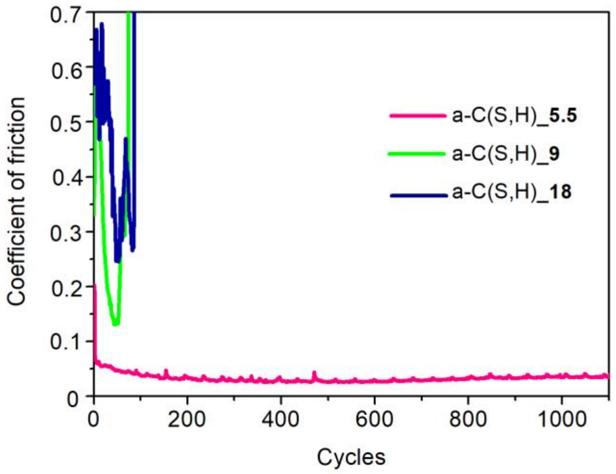

Figure S8. Characteristic evolution of the friction coefficient as a function of the cycle number for a-C(S,H) thin film coatings obtained by reactive PLD at the pressures of H_2S gas of 5.5, 9, and 18 Pa. Pin-on-disk tribometer testing was conducted in dry friction conditions (air+Ar mixture, RH~8%) at 22 °C.

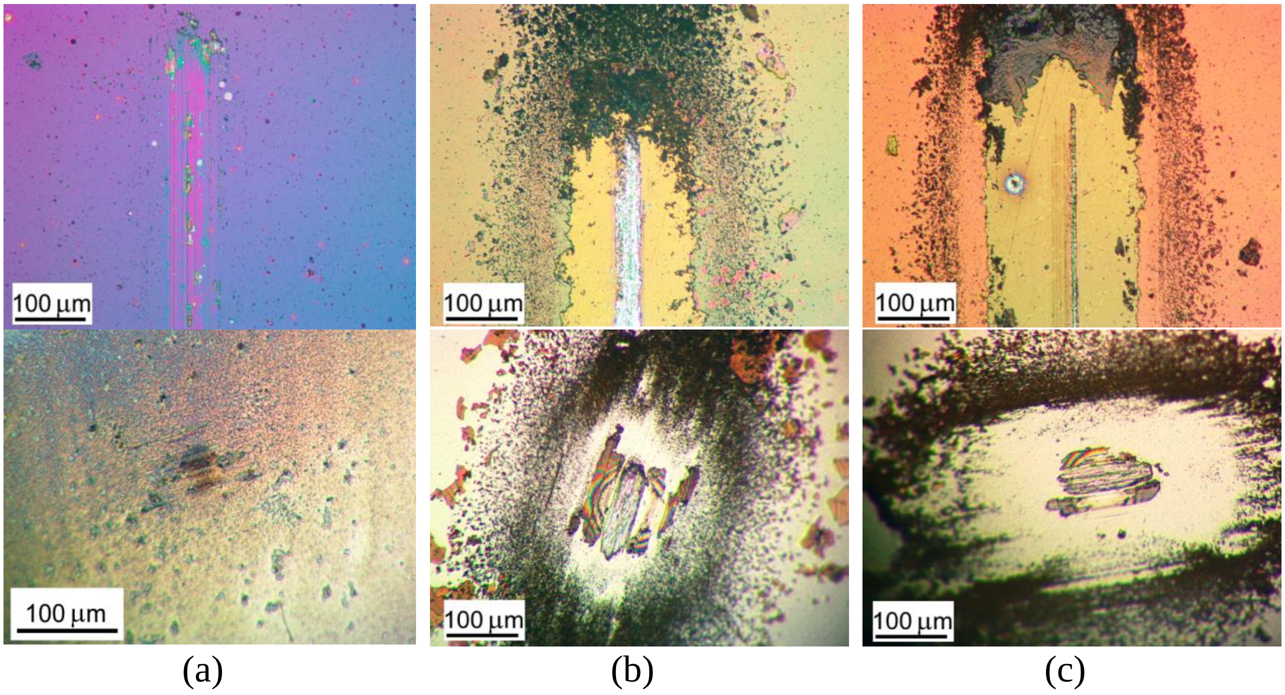

Figure S9. Optical images of wear tracks and wear scars formed on the steel substrates and steel balls for a-C(S,H) thin film coatings obtained by reactive PLD at the different pressures of H_2S gas: (**a**) 5.5, (**b**) 9, and (**c**) 18 Pa. Pin-on-disk tribometer testing was conducted in dry friction conditions at 22 °C. The test durations are indicated in Figure S8.

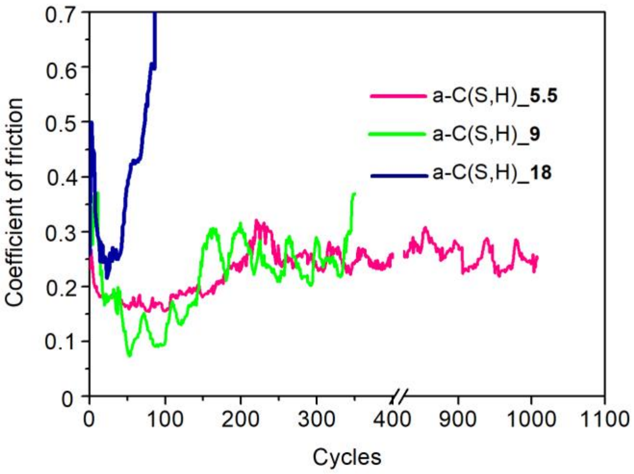

Figure S10. Characteristic evolution of the friction coefficient as a function of the cycle number for a-C(S,H) thin-film coatings obtained by reactive PLD at the pressures of H_2S gas of 5.5, 9 and 18 Pa. Pin-on-disk tribometer testing was conducted in dry friction conditions at −100 °C.

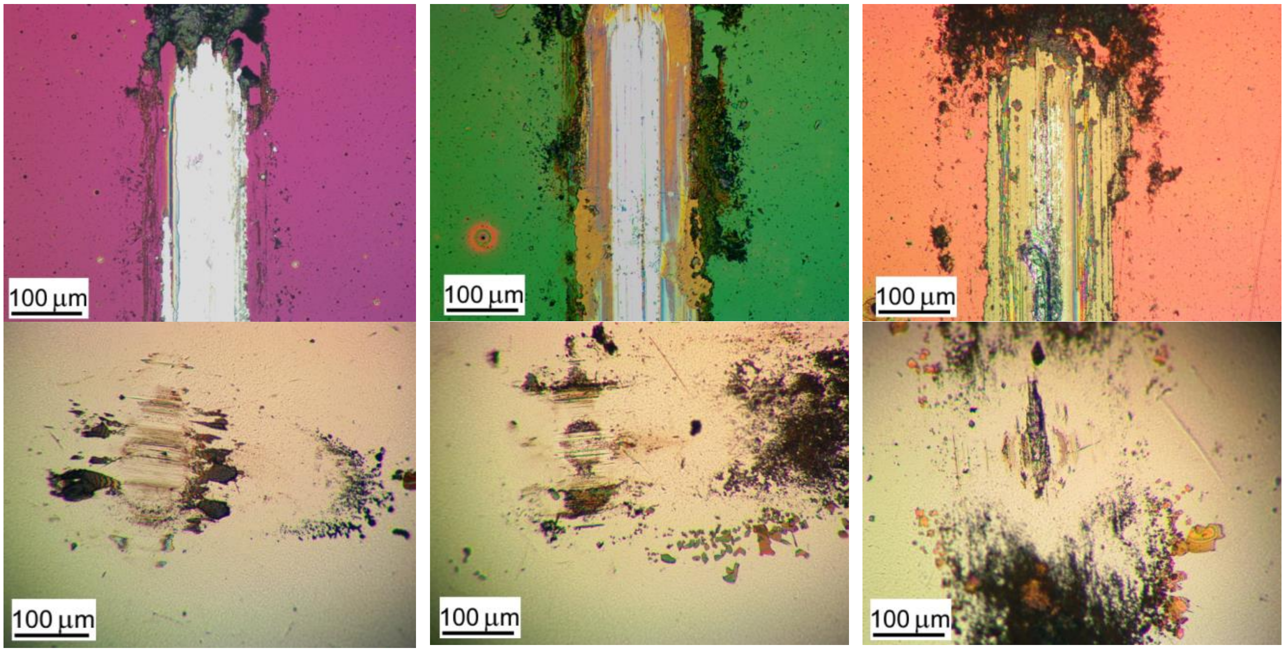

(a) (b) (c)

Figure S11. Optical images of wear tracks and wear scars formed on the steel substrates and steel balls for a-C(S,H) thin-film coatings obtained by reactive PLD at the different pressures of H_2S gas: (**a**) 5.5, (**b**) 9, and (**c**) 18 Pa. Pin-on-disk tribometer testing was conducted in dry friction conditions at −100 °C. The test durations are indicated in Figure S10.
